# Overexpression of Water-Responsive Genes Promoted by Elevated CO_2_ Reduces ROS and Enhances Drought Tolerance in *Coffea* Species

**DOI:** 10.3390/ijms24043210

**Published:** 2023-02-06

**Authors:** Isabel Marques, Isabel Fernandes, Octávio S. Paulo, Dora Batista, Fernando C. Lidon, Fábio Partelli, Fábio M. DaMatta, Ana I. Ribeiro-Barros, José C. Ramalho

**Affiliations:** 1 Plant-Environment Interactions and Biodiversity Lab (PlantStress & Biodiversity), Forest Research Centre (CEF), Instituto Superior de Agronomia (ISA), Universidade de Lisboa, 1349-017 Lisboa, Portugal; 2 Associate Laboratory TERRA, Instituto Superior de Agronomia (ISA), Universidade de Lisboa, 1349-017 Lisboa, Portugal; 3 cE3c—Center for Ecology, Evolution and Environmental Changes and CHANGE—Global Change and Sustainability Institute, Faculdade de Ciências, Universidade de Lisboa, 1749-016 Lisboa, Portugal; 4 Linking Landscape, Environment, Agriculture and Food (LEAF), Instituto Superior de Agronomia (ISA), Universidade de Lisboa, 1349-017 Lisboa, Portugal; 5 Unidade de Geobiociências, Geoengenharias e Geotecnologias (GeoBioTec), Faculdade de Ciências e Tecnologia (FCT), Universidade NOVA de Lisboa (UNL), 2829-516 Caparica, Portugal; 6 Centro Universitário do Norte do Espírito Santo (CEUNES), Departmento Ciências Agrárias e Biológicas (DCAB), Universidade Federal Espírito Santo (UFES), São Mateus 29932-540, ES, Brazil; 7 Departamento de Biologia Vegetal, Universidade Federal Viçosa (UFV), Viçosa 36570-900, MG, Brazil

**Keywords:** ABA signaling, coffee, functional analysis, ROS, stress, tolerance

## Abstract

Drought is a major constraint to plant growth and productivity worldwide and will aggravate as water availability becomes scarcer. Although elevated air [CO_2_] might mitigate some of these effects in plants, the mechanisms underlying the involved responses are poorly understood in woody economically important crops such as *Coffea*. This study analyzed transcriptome changes in *Coffea canephora* cv. CL153 and *C. arabica* cv. Icatu exposed to moderate (MWD) or severe water deficits (SWD) and grown under ambient (aCO_2_) or elevated (eCO_2_) air [CO_2_]. We found that changes in expression levels and regulatory pathways were barely affected by MWD, while the SWD condition led to a down-regulation of most differentially expressed genes (DEGs). eCO_2_ attenuated the impacts of drought in the transcripts of both genotypes but mostly in Icatu, in agreement with physiological and metabolic studies. A predominance of protective and reactive oxygen species (ROS)-scavenging-related genes, directly or indirectly associated with ABA signaling pathways, was found in *Coffea* responses, including genes involved in water deprivation and desiccation, such as protein phosphatases in Icatu, and aspartic proteases and dehydrins in CL153, whose expression was validated by qRT-PCR. The existence of a complex post-transcriptional regulatory mechanism appears to occur in *Coffea* explaining some apparent discrepancies between transcriptomic, proteomic, and physiological data in these genotypes.

## 1. Introduction

Drought events have become more frequent, severe, and erratic nowadays, affecting the quality and yield of most crops [1,2]. Under the initial stages of drought, stomatal closure usually occurs to reduce the loss of water through transpiration, but at the same time, decreasing the entrance of CO_2_ into leaves, limiting photosynthesis, and ultimately plant growth [3]. With an increase in the severity of drought, the functioning of photosynthesis can be further impaired by photochemical and biochemical dysfunctions occurring in pigments, photosystems performance, enzyme activities (namely RuBisCO), and cell membrane integrity [4,5,6,7,8]. The reduction in the photochemical energy use will also impose a secondary stress associated with the generation of reactive oxygen species (ROS) that oxidize, impair, and damage multiple cellular components, ultimately causing cell death [9].

In some C3 plants, the harsh effects of drought are sometimes counteracted by elevated [CO_2_] (eCO_2_), associated with a direct stimulation of photosynthesis, a reduction in stomatal conductance [10,11], and the strengthening of defense mechanisms and photosynthetic components, which altogether contribute to the preservation of the photosynthetic performance [12]. However, the positive effects of eCO_2_ in attenuating the impact of drought depend on the stress severity and its duration, and also on the species/genotypes involved [13]. For instance, eCO_2_ reduced the negative effects that drought imposed on the quality of sorghum grains by delaying physiological and metabolic responses to this stress [14]. In contrast, in soybean, eCO_2_ did not counteract the negative impacts of drought on photosynthesis and yield, and the minor benefits that were initially observed were progressively lost with increasing drought severity [15]. This highlights the potential for complex interactions among the abiotic factors of global change, which have been poorly investigated in woody plants, despite the urgency to develop adaptive strategies considering the future climate conditions.

Coffee is one of the most important agricultural commodities worldwide, generating about USD 200.0000 million [16], and constituting a crucial source of income for 20–25 million smallholder farmers, which are mostly based in the tropical region [17,18]. Despite the recognized resilience and metabolic flexibility of some coffee genotypes to environmentally stressful conditions, adverse temperatures and limited water availability are the major causes of crop failure, affecting the yield and quality of coffee beans, promoting livelihood insecurity, and constraining the value chain of coffee [19,20]. *Coffea arabica* is native to Ethiopian tropical forests at altitudes of 1600–2800 m, with an annual average of about 20 °C, whereas *C. canephora* is native to the lowland forests of the Congo River basin, growing from sea level up to 1200 m and under average temperatures between 24 and 26 °C, although without large oscillations. Currently, the optimum mean annual temperature range for arabica coffee is considered to be between 18 and 21 °C, although elite cultivars under intensive management allow the spread of arabica coffee to regions with average temperatures as high as 24–25 °C. *Coffea canephora* can grow under higher temperatures with optimum annual mean temperatures ranging from 22 to 30 °C, depending on authors [18]. Despite drought is a concern for the crop, some genotypes can maintain high photosynthetic rates, especially under eCO_2_ conditions [12,21], reducing physiological constraints imposed by drought (e.g., overcoming diffusional CO_2_ limitations due to stomatal closure), and reinforcing some defense mechanisms, contributing to maintaining photosynthetic performance and, likely, crop yield, at least under moderate levels of drought [12,17,22,23,24].

Here, we explore the underlying transcriptomic mechanisms by which coffee genotypes adjust to increasing drought severity and how eCO_2_ can modify such adjustments. Based on the fact that eCO_2_ improves resilience to drought stress at the physiological and biochemical levels [12], we hypothesized that eCO_2_ interacts at the transcriptomic level to promote a greater metabolic performance, and acclimation mechanisms, namely at the photosynthetic level. To test these hypotheses, we assessed the impacts of drought on two genotypes from the most important coffee-traded species, *Coffea canephora* Pierre ex A. Froehner cv. Conilon Clone 153 (CL153) and *C. arabica* L. cv. Icatu Vermelho (Icatu), grown under ambient (aCO_2_) or elevated (eCO_2_) air [CO_2_], and gradually subjected to moderate water deficit (MWD) or severe water deficit (SWD) conditions in comparison with well-watered (WW) plants. CL153 is a late maturation/ripening diploid clonal variety created from Emcapa 8131 (also known as Vitória 13) by Incaper (Vitória, ES, Brazil) that already showed some relevant drought tolerance while Icatu is an introgressed tetraploid variety originated from a cross between *C. canephora* and *C. arabica* cv. Bourbon Vermelho that was further crossed with *C. arabica* cv. Mundo Novo by IAC. The two genotypes display a relevant response ability to drought, although having different degrees of resilience as CL153 suffers a higher negative impact than Icatu in the photochemical and biochemical components of C-assimilation under severe drought [12,25]. Here, we explore the molecular mechanisms beyond such striking physiological and biochemical differences. Understanding the molecular mechanisms that ultimately determine the response of *Coffea* to climatic changes is crucial to mitigate their harmful effects and establish better scenarios to maintain the sustainability of the value chain of coffee.

## 2. Results

### 2.1. Overview of the RNA-Seq Data from the Two Coffee Genotypes

RNA sequencing resulted in an average of 21.8 million paired-end reads per sample, generating an average of 16.9 and 19.3 million high-quality unique reads in Icatu and CL153, respectively (Table S2). A high proportion of reads was mapped to the corresponding reference genome since only an average of 16% and 14% of reads from Icatu and CL153, respectively, were not mapped. Statistical details for each replicate are depicted in Table S2.

**Figure 1:**
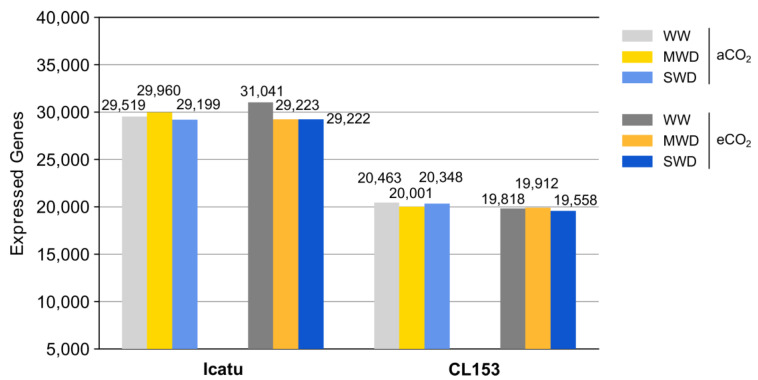
Total number of expressed genes in Icatu and CL153 plants grown under different watering conditions (well-watered, WW; moderate water deficit, MWD; and severe water deficit, SWD), and under ambient air 380 μL L^−1^ [CO_2_] (aCO_2_) or elevated 700 μL L^−1^ [CO_2_] (eCO_2_), at 25/20 °C.

The number of expressed genes varied in Icatu from 29199 (SWD-aCO_2_) to 31041 (WW-eCO_2_), while much lower values were found in CL153, ranging from 19558 (SWD-eCO_2_) to 20463 (WW-aCO_2_) (Figure 1). A principal component analysis (PCA) based on gene expression generally clustered the samples according to the different water treatments (Figure S1). In addition, MWD plants were usually closer to WW plants under eCO_2_ but with SWD plants under aCO_2_ conditions, especially when considering Icatu plants (Figure S1).

### 2.2. Response of Differentially Expressed Genes (DEGs) to Drought and eCO_2_

**Figure 2:**
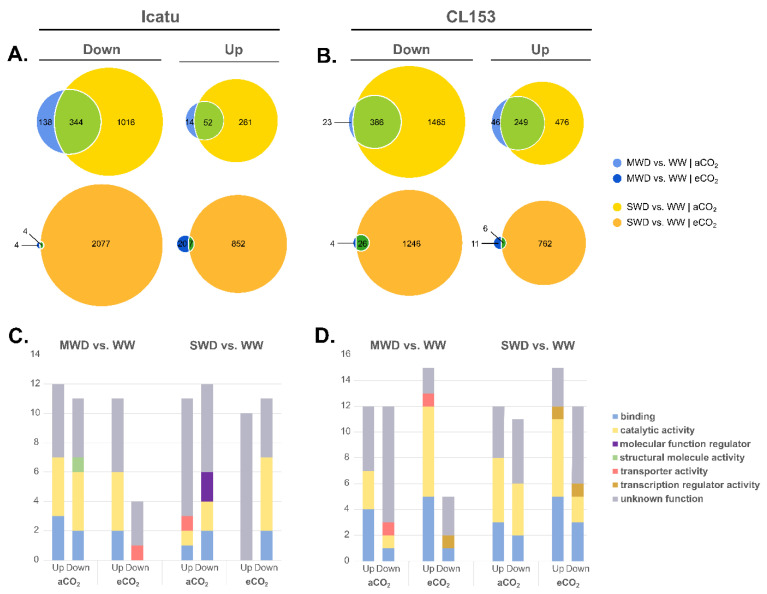
Patterns of differentially expressed genes (DEGs) at MWD or SWD in comparison with WW in (**A**) Icatu and (**B**) CL153 plants grown under either aCO_2_ (light colors) or eCO_2_ (dark colors), at 25/20 °C (day/night). Blue: DEGs specifically found under MWD. Yellow: DEGs specifically found under SWD. Green: DEGs expressed by both water conditions. Gene ontology (GO) terms found among the top up- and down-regulated differentially expressed genes (DEGs) in (**C**) Icatu and (**D**) CL153 plants. GO terms were selected according to UniProtKB and QuickGO databases.

The impact of MWD on the total number of DEGs was minimal under eCO_2_ in comparison with plants under aCO_2_ (Figure 2A,B). The number of DEGs commonly triggered by both water deficits was much lower under eCO_2_ than under aCO_2_. In fact, under MWD, a high number of DEGs were up-regulated under eCO_2_ plants when compared to their aCO_2_ counterparts, especially in Icatu.

The harshest drought level triggered a high down-regulation of DEGs, in the two genotypes, independently of [CO_2_] levels (Figure 2A,B; Table S3). The strong impact of the SWD was also depicted on heatmaps visualizing the expression profile of all significant DEGs, where a high degree of variation was found under this harsher drought level in contrast with MWD, which promoted only minor effects (Figure S2). The full list of DEGs can be found in Tables S4 and S5.

The majority of the up- and down-regulated DEGs found had no annotated functions (Figure 2C,D). The remaining ones were mostly involved in ‘catalytic activities’, followed by ‘binding’ in the two genotypes regardless of [CO_2_] levels (Figure 2C,D). Under aCO_2_, MWD triggered the down-regulation of DEGs involved in ‘structural molecule activity’ in Icatu. Instead, in CL153 ‘transporter activity’ DEGs were down-regulated under aCO_2_ but up-regulated under eCO_2_. Still, under MWD and eCO_2_ there was a down-regulation of DEGs involved in ‘transporter activity’ in Icatu and ‘transcription regulator activity’ in CL153.

Under harsher drought conditions (SWD), Icatu aCO_2_-plants showed an up-regulation of DEGs involved in ‘transporter activity’, ‘catalytic activity’, and ‘binding’, but the latter two categories were involved in down-regulated DEG, together with molecular function regulators (Figure 2C,D). CL153 plants under SWD and aCO_2_ showed a high number of up- and down-regulated DEGs associated with catalytic activity and binding functions. Under eCO_2_, while Icatu plants showed a down-regulation of DEGs involved in catalytic activities and binding, CL153 plants rather showed up- and down-regulated DEGs involved in these two categories, as well as in transcription regulator activities.

### 2.3. Drought and eCO_2_ Impact on DEGs Associated with Specific Biochemical Pathways

**Figure 3:**
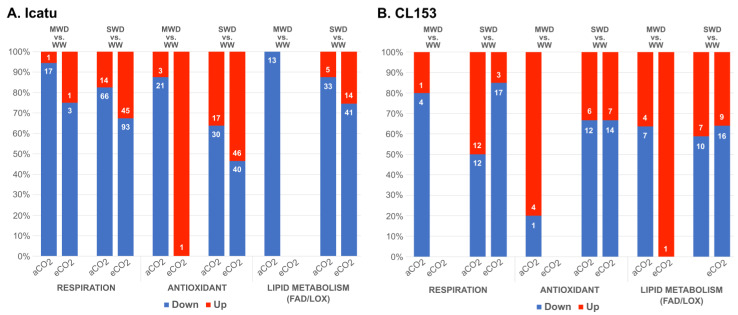
Proportion and number of significantly up- (red) and down-regulated (blue) DEGs associated with physiological and biochemical responses found in (**A**) Icatu and (**B**) CL153 plants grown under MWD or SWD in comparison with WW plants grown under aCO_2_ or eCO_2_, at 25/20 °C (day/night).

Drought had a relevant impact on a high number of DEGs involved in respiration, antioxidant, and lipid biochemical pathways, especially in Icatu where a total of 342 DEGs (respiration: 168, antioxidant: 106, lipid metabolism: 68 DEGs; Table S6) were found to be affected, in comparison with 91 in CL153 (respiration: 31, antioxidant: 29, lipid metabolism: 31 DEGs; Table S7). Under MWD and aCO_2_, DEGs associated with these biochemical pathways were mostly down-regulated but they were substantially reduced under eCO_2_ (Figure 3). The positive effect of eCO_2_ was even more relevant under SWD in Icatu plants, which showed an increase in up-regulated DEGs.

**Figure 4:**
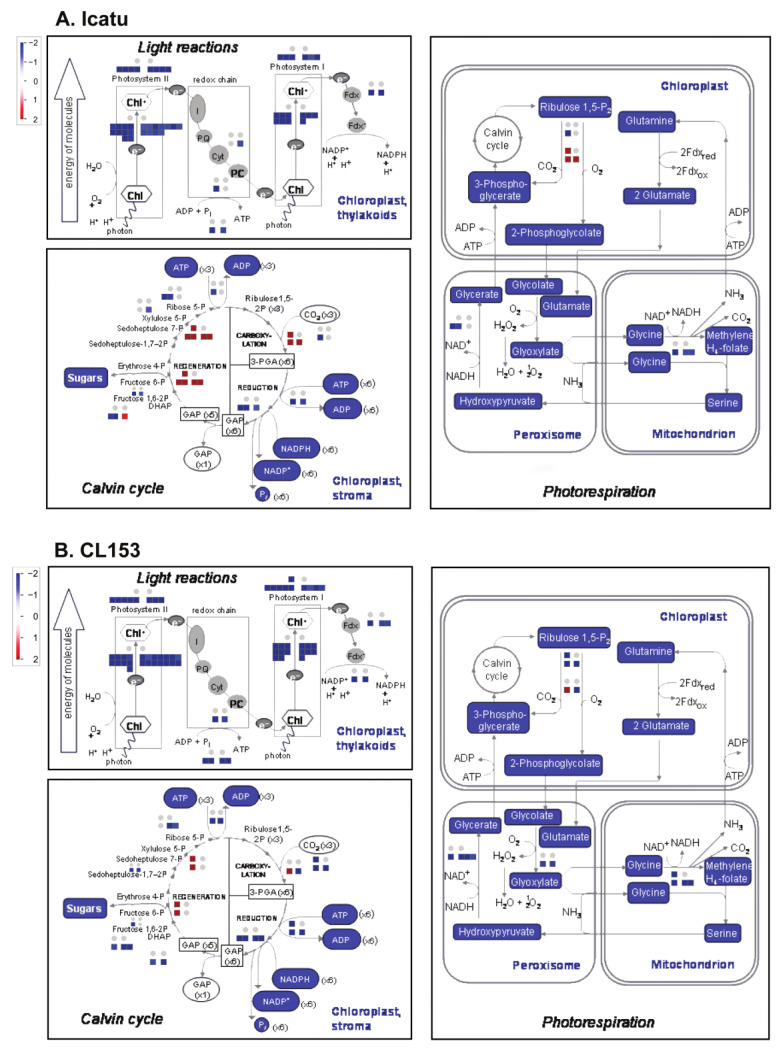
Changes in the expression of photosynthesis-related DEGs in (**A**) Icatu and (**B**) CL153 plants grown under MWD or SWD in comparison with WW plants grown under aCO_2_ or eCO_2_, at 25/20 °C (day/night). Pathway diagrams of light reactions of photosynthesis, the Calvin cycle, and photorespiration with superimposed color-coded squares drawn in MapMan. The color scale represents the level of regulation (blue: down-regulated DEGs; red: up-regulated DEGs). Results in the squares are presented in the following order: top left—MWD-aCO_2_; top right—MWD-eCO_2_; bottom left—SWD-aCO_2_; bottom right—SWD-eCO_2_. Grey dots represent an absence of DEGs. An absence of data in eCO_2_ indicates the absence of DEGs.

DEGs related to light reactions of photosynthesis, the Calvin cycle, and photorespiration visualized through MapMan showed minor effects under MWD while under SWD they were predominantly down-regulated, independently of [CO_2_] (Figure 4). No photosynthetic-related DEGs were found under MWD and eCO_2_ either in Icatu (Table S8) or CL153 (Table S9). The expression of the remaining photosynthetic-related DEGs decreased with drought with a few exceptions: transkelotase and RuBisCO activase 1 were up-regulated in Icatu plants in both water deficits (Table S8); in CL153 plants, the translocase was up-regulated in both water deficits but only under aCO_2_; while RuBisCO activase 1 was up-regulated under SWD and aCO_2_ but down-regulated under SWD and eCO_2_ (Table S9).

### 2.4. DEGs Involved in the Response to Water Deprivation and Desiccation

**Figure 5:**
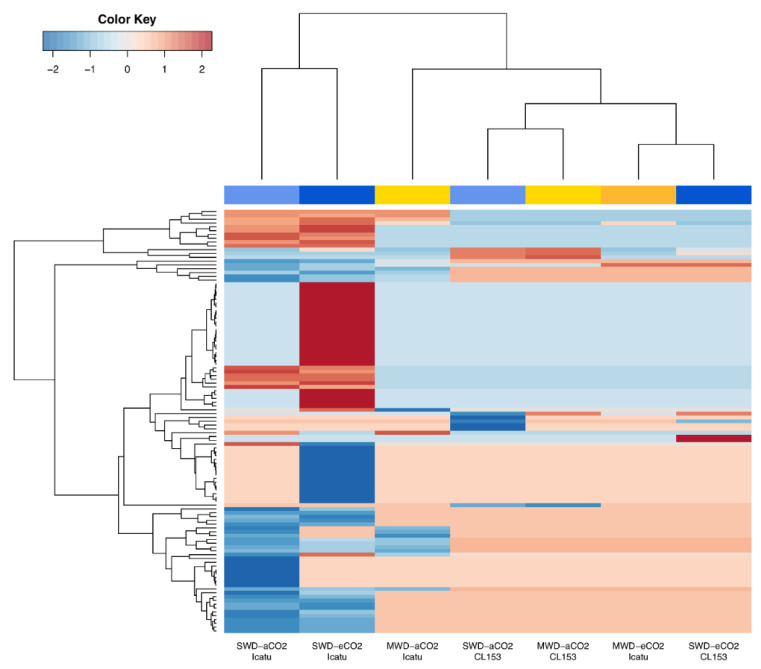
Clustered heatmaps and dendrograms of the normalized log_2_ fold change (FC) visualizing the expression of drought-related DEGs in Icatu and CL153 plants grown under MWD or SWD in comparison with WW plants grown under aCO_2_ or eCO_2_, at 25/20 °C (day/night). Values were scaled by row using Z-scores. Hot colors represent up-regulated DEGs, and cold colors represent down-regulated DEGs. Column color labels groups comparisons by water treatments (yellow/orange: MWD; light blue/dark blue: SWD; light colors represent aCO_2_; dark colors represent eCO_2_). An absence of data in CL153 plants under MWD-eCO_2_ indicates the absence of significant DEGs.

Drought triggered a high number of DEGs involved in water deprivation and desiccation responses, especially in Icatu plants (155 DEGs) in comparison with CL153 (16 DEGs). The full list of DEGs is depicted in Table S10. DEGs involved in water-deprivation responses showed minor changes under MWD (Figure 5). For instance, no significant DEGs were detected in CL153 plants grown under MWD and eCO_2_. Under SWD, most drought-responsive genes were down-regulated under aCO_2_ but up-regulated under eCO_2_, an effect mostly seen in Icatu plants (Figure 5).

Icatu plants grown under MWD and aCO_2_ showed the most down-regulation for the PLAT domain-containing protein 3-like (FC: −7.32) and the most up-regulation for the HVA22-like protein e (FC: 4.94), while under eCO_2_, only one DEG was found: the galactinol synthase 2-like (FC: 3.45; Table 1 and Table S10). SWD and aCO_2_ triggered the most down-regulation of the probable xyloglucan endotransglucosylase/hydrolase protein 6 (FC: −12.31) and the most up-regulation of the protein phosphatase 2C 51-like (FC: 5.62). Under eCO_2_ and SWD, the lowest down-regulation was also found for the probable xyloglucan endotransglucosylase/hydrolase protein 6 (FC: −11.17), while the highest up-regulation was recorded for the Late Embryogenesis Abundant protein Dc3-like (*LEA-DC3*; FC: 7.19). Notably, a high number of DEGs were involved in the antioxidant system as the galactinol synthase 2-like, sucrose synthase 2-like, the homeobox-leucine zipper *ATBH-12*, as well as aquaporins were up-regulated in Icatu under SWD and eCO_2_ (Table S10).

By contrast, the minor responses recorded in CL153 plants and mostly recorded under SWD and aCO_2_ involved a high number of Aspartic Protease in Guard Cell 1-like (*ASPG1*; 6 out of 16 total DEGs; Table 2). In this genotype, the 18 kDa seed maturation was the most up-regulated under MWD, independently of CO_2_ levels (aCO_2_ FC: 9.18; eCO_2_ FC: 8.79) while the lowest was the *APG1*, also independently of CO_2_ levels (aCO_2_ FC: −7.93; eCO_2_ FC: −8.16; Table 2). Remarkably, under aCO_2_, the dehydrin *DH1a* was also highly up-regulated under both water deficit levels (FC: 6.05 for MWD; FC 5.47 for SWD) while it was less expressed under eCO_2_ and only under SWD (FC 2.37). In fact, under eCO_2_ and MWD, no water-deprivation-related DEG was recorded. Under SWD, only one down-regulated DEG was found (the putative movement binding protein 2C; FC:−19.92) while the remaining were up-regulated, especially the *APG1* (FC: 3.96).

### 2.5. Enriched Gene Ontology (GO) Terms and Functional Pathways Responding to Drought and eCO_2_

**Figure 6:**
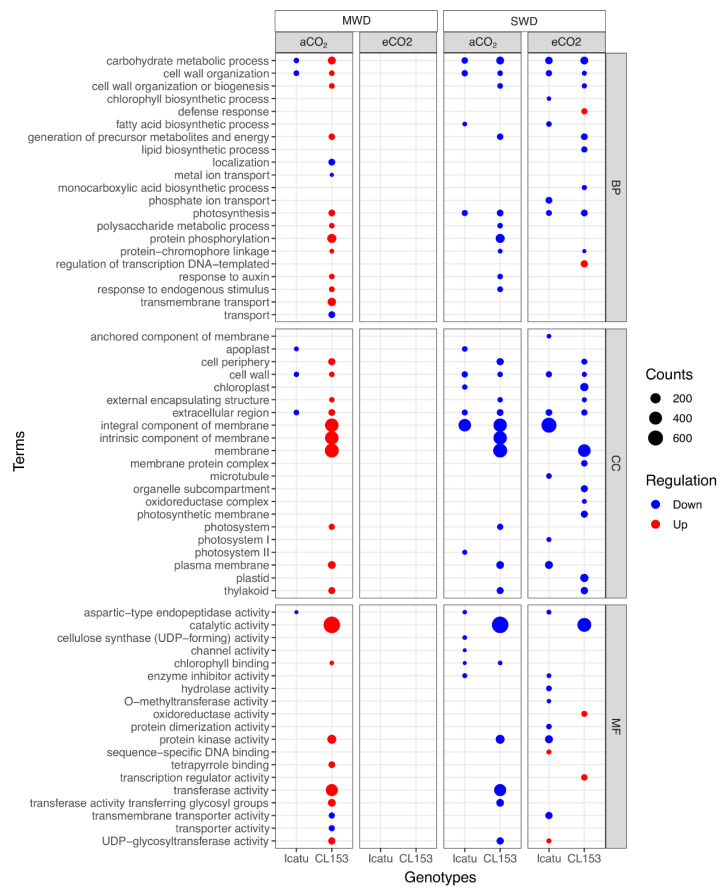
Over-representation analysis of gene ontology (GO) terms found among Icatu and CL153 DEGs performed with gProfiler against the functional annotation of *Coffea arabica* and *Coffea canephora* genomes, respectively. Significantly (g:SCS < 0.01) enriched GO was ranked by decreasing log_2_ fold-change (FC), considering the effect of MWD or SWD in comparison with WW plants grown under aCO_2_ or eCO_2_, at 25/20 °C (day/night). Dot plots with all GO terms were filtered by REVIGO with similarity = 0.5, and a count > 10 cut-offs. Terms are grouped by the main category: Biological Process (BP), Molecular Function (MF), and Cellular Component (CC). Counts (size) indicate the number of DEGs annotated with each GO term and color represents the type of regulation (blue: down-regulated DEGs; red: up-regulated DEGs). An absence of data in eCO_2_ under MWD indicates the absence of enriched GO terms.

Icatu plants under aCO_2_ showed a moderate increase in enriched GO terms as water deficit severity increased, being all associated with down-regulated DEGs (Figure 6). eCO_2_ attenuated MWD impact and no enriched categories were found under this drought level in Icatu plants, while under SWD, Icatu plants showed a high number of enriched GO terms mostly linked to down-regulated DEGs. From all enriched categories, the greatest effect was recorded under SWD in the ‘integral component of the membrane’ (GO:0016021), independently of [CO_2_] (Figure 6; Table S11). However, under SWD and eCO_2_, two enriched categories were linked to up-regulated DEGs: ‘sequence-specific DNA binding’ (GO:0043565) and the ‘UDP−glycosyltransferase activity’ (GO:0008194).

By contrast, drought triggered a higher number of enriched GO terms in CL153 than in Icatu (84 vs. 43) (Figure 6; Table S12). Under aCO_2_ and MWD, CL153 plants were enriched in GO terms mostly involved up-regulated DEGs, except ‘localization’ (GO:0051179), ‘metal ion transport’ (GO:0030001), ‘transport’ (GO:0006810), ‘transmembrane transporter activity’ (GO:0022857), and ‘transporter activity’ (GO:0005215). The opposite was recorded in SWD plants under aCO_2_, where all enriched categories were linked to down-regulated DEGs. By contrast, CL153 plants under eCO_2_ showed no enriched categories under MWD (as reported also for Icatu plants), while under SWD they were mostly linked to down-regulated DEGs, except ‘defense response’ (GO:0006952), ‘regulation of transcription DNA-templated’ (GO:0006355), ‘oxidoreductase activity’ (GO:0016491) and ‘transcription regulator activity’ (GO:0140110). CL153 plants under aCO_2_ showed an enrichment in many down-regulated categories involving the cellular membrane, which were less recorded under eCO_2_ (Figure 6). Remarkably, the ‘catalytic activity’ (GO:0003824), which was up-regulated under MWD and aCO_2_, was down-regulated under SWD, regardless of [CO_2_] (Figure 6).

**Figure 7:**
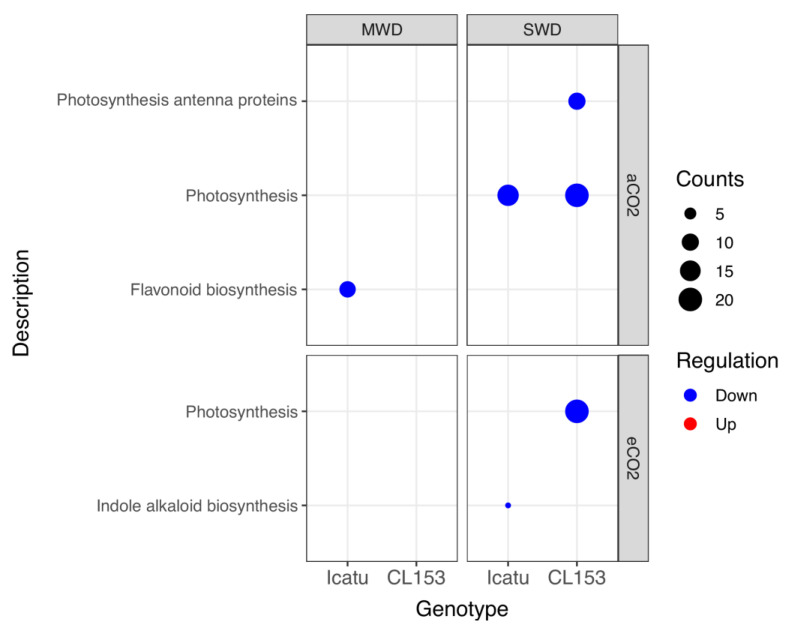
Over-representation analysis of KEGG pathways of Icatu and CL153 down-regulated DEGs performed with gProfiler, using the DEGs’ *Arabidopsis thaliana* homologs mapped through blastx, against the functional annotation of its reference genome. Significantly (g:SCS < 0.01) enriched KEGG pathways of DEGs ranked by decreasing log_2_ fold-change (FC), considering the effect of MWD or SWD in comparison with WW plants grown under aCO_2_ or eCO_2_, at 25/20 °C (day/night). Counts (size) indicate the number of DEGs annotated with each GO term. An absence of data in CL153 indicates the absence of significant KEGG pathways.

Based on the Kyoto Encyclopedia of Genes and Genomes (KEGG), only pathways linked to down-regulated DEGs were found to be significantly affected by drought (Figure 7). Under MWD and aCO_2_, the ‘flavonoid biosynthesis’ was the only KEGG pathway affected by drought and only in Icatu plants. Under SWD, the ‘photosynthesis’ pathway was mostly affected in Icatu and CL153 under aCO_2_, but only under eCO_2_ in CL153 plants. In addition, the ‘indole alkaloid biosynthesis’ pathway was affected under eCO_2_ in Icatu plants, while in CL153 plants, the ‘photosynthesis antenna proteins’ pathway was also affected under aCO_2_ (Figure 7).

### 2.6. Validation of RNA-Seq Results by qRT-PCR

**Figure 8:**
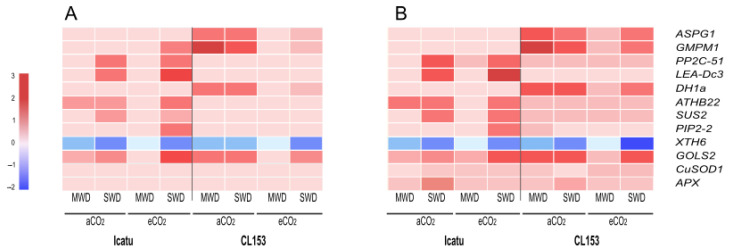
Heatmap of expression levels (log_2_ ratio) of the selected genes obtained from RNA-seq (**A**) and validated by qRT-PCR (**B**), considering the effect of MWD or SWD in comparison with WW-Icatu and CL153 plants, grown under aCO_2_ (light colors) or eCO_2_ (dark colors), at 25/20 °C (day/night). *GMPM1*: 18 kDa seed maturation; *PP2C-51*: protein phosphatase 2C 51-like; *LEA-DC3*: late embryogenesis abundant protein Dc3-like; *DH1a*: dehydrin DH1a; *ATHB22*: homeobox leucine zipper; *SUS2*: sucrose synthase 2-like; *PIP2-2*: aquaporin PIP2-2-like; *XTH6*: xyloglucan endotransglucosylase/hydrolase protein 6; *GOLS2*: galactinol synthase 2-like; *CuSOD1*: superoxide dismutase [Cu-Zn]; *APX_Chl_*: chloroplast ascorbate peroxidase.

All transcripts associated with a differential gene expression (*ASPG1, GMPM1, PP2C-51, LEAD-C3, DH1a, ATHB22, SUS2, PIP2-2, XTH6, GOLS2, CuSOD1, APX_Chl_*) in the RNA-seq workflow showed a change in the same direction under qRT-PCR, revealing a general agreement between RNA-seq sequencing and qRT-PCR analysis (Figure 8). Except for *XTH6*, all analyzed transcripts involved in the regulation or protection against water deficit were up-regulated under drought conditions, especially under SWD and eCO_2_.

## 3. Discussion

### 3.1. Differential Transcriptional Drought Regulation Responses in the Two Coffea Genotypes

Despite the information available, the molecular basis of stress responses in woody species is still limited in comparison with model plants. Understanding which genes may play a role under different degrees of drought severity, and which physiological traits are controlled by such genes, is of crucial importance for understanding the capabilities of trees to successfully cope with stress, especially in commercially important crops such as coffee. To maintain the global supply of this crop, it is important to promote the screening and development of tolerant varieties to face the increasingly expected impacts of drought events. In this study, we showed that the two genotypes, belonging to two different *Coffea* species, activated distinct transcriptional responses to cope with drought.

In comparison with CL153, Icatu showed (i) a higher number of expressed genes under MWD, but especially under SWD (Figure 1); (ii) a higher number of specific DEGs, especially under SWD (Figure 2); (iii) a higher number of DEGs involved in respiration, antioxidant activities, and lipid metabolism (Figure 3); (iv) a higher activation of DEGs involved in light reactions of photosynthesis, the Calvin cycle and photorespiration (Figure 4); and (v) a higher number of DEGs involved in responses to water deprivation and desiccation (Figure 5). This transcriptional result explains previous physiological studies that reported a higher photochemical performance in Icatu than in CL153, due to the preservation or reinforcement of photosynthetic components, strengthened enzymatic antioxidative system, and a greater abundance of photoprotective pigments [7,12,26,27].

Protein phosphatases (*PP2Cs*) were found to be involved in Icatu responses to drought (*PP2C 51-like*; Table 1). *PP2Cs* are a class of evolutionarily conserved serine/threonine protein phosphatases involved in stress responses and have been implicated in abscisic acid (ABA) signal transduction [28]. Transgenic studies suggest that many *PP2Cs* participate in drought responses, negatively regulating ABA signaling pathways in *Arabidopsis* [29,30], tomato [31], *Populus euphratica* [32], and *Artemisia annua* [33].

In response to drought, CL153 rather activated a high number of aspartic proteases (*ASPG1*; Table 2). Aspartic proteases play a fundamental role in the response of plants to drought, especially during ROS increments [34]. ROS can act as a positive regulator of ABA signaling in guard cells, but the excessive accumulation of ROS during stress can be toxic [35]. Thus, to balance ROS production and scavenging, either the levels are modulated through signal transduction, or the cells have mechanisms to detoxify excessive ROS during stress [36]. Hence, the up-regulation of aspartic proteases in CL153 may help to scavenge the excessive amount of ROS. The up-regulation of dehydrins (namely *DH1a*) in both water deficits may also be involved in protective reactions to dehydration [37]. Dehydrins were also found to be highly expressed in the leaves of drought-stressed *Coffea* plants such as *C. arabica* cvs. Catuaí and Mundo Novo, *C. canephora* cv. Apoatã [38], as well as in the genotypes studied here [39], where they seem to play a key role in the acclimation response of *Coffea*.

Our transcriptomic results also found a high number of DEGs involved in the antioxidant system, especially in Icatu (Figure 3). This suggests the action of three mechanisms involved in drought acclimation: (i) activation of antioxidant activities to scavenge the excessive amount of ROS and reduce oxidative damage in plants, including enzymes (e.g., catalase, superoxide dismutases, peroxidases) and non-enzymatic molecules (e.g., ascorbic acid, α-tocopherol, raffinose family oligosaccharides), in agreement with studies from other plants [40,41]; (ii) antioxidative mechanisms complemented with thermal dissipation mechanisms (e.g., photoprotective carotenoids) and changes in the cyclic electron flow (CEF) to protect the photosystem (PS) I and/or II. In fact, (i) and (ii) are transversally found in resilient *Coffea* genotypes as response mechanisms to drought [12,26], heat [42,43,44], cold [45,46], and high irradiance [47,48] stresses. Additionally, the activation of other molecules, such as aquaporins reported here in Icatu and in previous studies involving other *C. arabica* genotypes [49] or the heat shock protein 70 kDa as reported also in other *Coffea* studies [39,50]. Altogether, results reveal the expression of DEGs, whose products are important to limit or control water loss in Coffea, regulating stomatal closure in leaves subjected to drought conditions. Their overexpression upon stress was validated by qRT-PCRs (Figure 8), supporting the action of several antioxidant molecules in response to drought. It also corroborates the results of RNA-sequencing, providing a set of candidate genes involved in drought tolerance in coffee plants. These mainly include genes encoding antioxidant enzymes involved in the biosynthesis of small antioxidant molecules and water and ion movement, such as aquaporins and ion transporters or the biosynthesis of osmolytes. Other proteins that function directly in the protection of proteins and membranes, such as late embryogenesis abundant proteins, heat shock proteins, protein phosphatases, and aspartic proteases also play important roles in Coffea resilience to abiotic stresses. This vast information will help in the identification of target sites suitable for gene editing, which will undoubtfully make progress in the future.

### 3.2. Influence of eCO_2_ in Drought-Responsive Genes

The level and type of transcripts found in this study support the positive effects of eCO_2_ in mitigating some impacts of drought, especially in the functioning and components of the photosynthetic apparatus [12]. The up-regulation of antioxidant-related DEGs under eCO_2_, mostly under SWD and in Icatu plants (Figure 3) is congruent with physiological measurements that showed an increase in Cu,Zn-SOD activity under MWD, rising further under SWD, but only under eCO_2_ [27]. The up-regulation of antioxidant-related DEGs under eCO_2_ might strengthen the scavenging potential for molecular O_2_ when the photochemical use of energy is small, reducing the probability of electron flow to lower energy states [12], helping to alleviate some impacts of drought in the photosynthetic machinery (Figure 4).

The imposition of eCO_2_ to SWD plants promoted the up-regulation of several important stress response genes, including the *LEA-DC3* in Icatu and the *APG1* in CL153 as mentioned before. Late embryogenesis abundant proteins compose the most abundant and characterized group of intrinsically disordered proteins that prevent and repair the damage caused by environmental stresses. A positive association between the accumulation of *LEA* and environmental stresses, such as drought, heat, and salinity, has been outlined in several other plant species, where they bind to enzymes to prevent the loss of activity under stressful conditions [51,52]. This is consistent with the biological functions of *LEAs* namely in oxidant scavenging activities, enzyme and nucleic acid preservations, and the membrane maintenance that occurs in genotypes/species that can better cope with environmental stresses [53].

Directly or indirectly, ABA signaling pathways, which include ABA-dependent, ABA-responsive element/ABRE-binding factors (ABRE/ABF), as well as ABA-independent genes as dehydration-responsive elements regulate *Coffea* responses to stress. ABA is involved in both drought-induced and eCO_2_-induced stomatal closure in a dual way, including root-derived and foliar ABA. For instance, under well-watered conditions, *C. arabica* plants grown under eCO_2_ showed lower whole-plant transpiration rates than under aCO_2_ [21]. These changes, although unrelated to stomatal conductance or foliar ABA levels, are associated with faster stomata closure rates upon rapid increases in vapor pressure deficit under eCO_2_ [21]. For instance, during exposure to drought, *Coffea* plants grown under eCO_2_ can maintain higher water potentials and plant hydraulic conductance than under aCO_2,_ due to a higher transcript abundance of aquaporins [21]. In the genotypes studied here, drought alone prompted gradual ABA increases of ~46% in MWD plants in both genotypes, and 100% (CL 153) and 184% (Icatu) under SWD conditions, whereas single eCO_2_ increased ABA levels (by 85%) but only in Icatu [12]. This is important since ABA controls stomatal closure under stress, which is one of the first defense responses to reduce water loss by restricting the transpiration flow in response to a rising air evaporative demand or a decreased soil water availability [54]. This suggests that, at least under MWD, eCO_2_ seemed to decouple ABA action from stomatal closure in both genotypes. In agreement with this hypothesis, dehydration is postponed in MWD plants under eCO_2_, and a delayed stomata response to soil drying under eCO_2_ is found in some coffee genotypes [12,21,22], maintaining greater stomatal conductance than expected based on ABA concentrations. Such a greater stomatal opening under MWD would allow greater C-assimilation gains under eCO_2_ [12], also revealing a more profound impact of eCO_2_ than the direct stimulation of C-assimilation. A better understanding of ABA concentrations in the xylem sap is necessary to understand the sensitivity of *Coffea* stomata to [ABA] xylem.

### 3.3. Overall Regulatory Mechanisms Involved in Coffea Responses

MWD had minor impacts under aCO_2_ with few enriched categories being found in Icatu, and all were associated with down-regulated DEGs, while CL153 plants showed a high increase in GO categories, and were mostly associated with up-regulated DEGs (Figure 6).

Under SWD, a few enriched categories were even up-regulated in Icatu (‘sequence-specific DNA binding’ and the ‘UDP−glycosyltransferase activity’) and CL153 (‘defense response’, ‘regulation of transcription DNA-templated’, ‘oxidoreductase activity’ and ‘transcription regulator activity’). Genes assigned to these GO terms are usually involved in a high number of developmental processes and stress responses, plant hormone activation, and the production of antioxidants in response to stresses, including drought [55]. In *Coffea*, as in other species, glycosylation catalyzed by glycosyltransferases, as well as oxidoreductase activities, play an essential role in regulating the stability, availability, and biological activity of antioxidant compounds and the integrity of cellular membranes [56]. This is crucial to cope with the effects of water deprivation in *Coffea* since tolerance is associated with the ability of tissues to withstand low water potentials and plant membrane transport systems play a significant role under water scarcity [57]. Depending on energy needs, translocation through biological membranes occurs, passively or actively, but drought can compromise the integrity of membranes and is, therefore, relevant an up-regulation of categories linked to cellular membranes in the two *Coffea* genotypes, even under MWD.

The ‘flavonoid biosynthesis’ KEGG pathway was affected in Icatu plants grown under MWD and aCO_2_ (Figure 7). Plant phenolic compounds, especially flavonoids, can provide resistance to biotic and abiotic stresses, and can be enhanced upon drought [58]. As non-enzymatic antioxidants, hydroxyl groups in flavonoids participate in the scavenging of oxygen free radicals, alleviating stress-induced oxidative damage as is widely reported [59], thus, affecting acclimation responses of Icatu, at least under aCO_2_. However, SWD affected the KEGG photosynthetic pathway, namely in Icatu plants under aCO_2_, as well as in CL153 plants under both [CO_2_] levels together with the ‘photosynthesis antenna proteins’ pathways in CL153 plants under SWD and aCO_2_ (Figure 7). Since these pathways are both linked to down-regulated DEGs, this would imply some effects in the photosynthetic machinery of these genotypes, especially in CL153. Indeed, a large dilution of the impacts of drought on the net photosynthesis in these genotypes was found to be promoted by eCO_2_ under MWD, consistent with a tendency to the maintenance of the PSII efficiency and higher PSs activity in both genotypes while under SWD, the net photosynthesis and stomatal conductance were severely reduced, regardless of [CO_2_] [12]. Nevertheless, even under SWD, a relevant potential for C-assimilation was preserved, with the photosynthetic capacity (A_max_) showing values close to 60% (CL153), or even higher than 70% (Icatu) relative to those displayed by their respective WW controls [12]. This photochemical protective mechanism results in a lower need for dissipation processes and a reduced PSII inhibition status [43] consistent with the down-regulation of photosynthetic-related DEGs found in this study (Figure 4) or the KEGG enrichment results (Figure 7) since no new molecules would be needed due to the protective mechanisms involved.

A minor inhibition in the ‘indole alkaloid biosynthesis’ (IAB) KEGG pathway was also recorded in Icatu under SWD and eCO_2_. Plant peroxidases may accept alkaloids as substrates, as well as phenols and flavonoids [60], and metabolize H_2_O_2_ as an electron donor for phenol peroxidases, resulting in the formation of phenoxyl radicals, which can be regenerated by a non-enzymatic reaction with an ascorbate function as an H_2_O_2_ scavenging system [61]. Thus, either the IAB KEGG pathway is not involved/not needed in acclimation responses of Icatu or this would suggest a decrease in the defense ability of this genotype against oxidative stress. However, that contrasts with the high activity found in protective molecules and antioxidant enzymes in Icatu [12,27], and the large reinforcement of Cu,Zn-SOD, APX, and catalase (CAT) activities [7].

### 3.4. Evidence of Post-Transcriptional Regulatory Mechanisms in Coffea Responses to Stress

Previous findings showed that *Coffea* plants, namely Icatu, can maintain the potential photosynthetic functioning under the imposition of SWD due to a greater antioxidative response, which contrasts with the transcriptomic results shown here where photosynthetic-related DEGs were mostly down-regulated (Figure 3 and Figure 4) and the KEGG photosynthetic pathway was highly affected under SWD (Figure 7). Physiological studies showed that increasing drought severity progressively affected gas exchange and fluorescence parameters in both genotypes, with non-stomatal limitations becoming gradually dominating, and having strong impacts on the photochemical and biochemical components and functioning of *Coffea*, especially in CL153 plants under SWD and aCO_2_ [12]. In contrast, Icatu plants were tolerant to SWD, with minor, if any, negative impacts on the potential photosynthetic functioning and components, e.g., A_max_, F_v_/F_m_, electron carriers, photosystems (PSs) and RuBisCO activity, under aCO_2_ [12]. Under MWD, eCO_2_ delayed stress severity and promoted photosynthetic functioning in both genotypes, with lower energy dissipation, while stomatal closure was decoupled from increases in ABA. Under SWD, most of the negative impacts felt on the photosynthetic components and their potential performance were reduced under eCO_2_, at least considering CL153, since Icatu was barely affected in both [CO_2_] levels under SWD [12]. Still, strong effects were detected in RuBisCO, as the most sensitive photosynthetic component [12]. However, proteomic analyses have also shown a higher abundance of drought-responsive proteins in Icatu than in CL153, together with enriched GO terms, and enriched KEGG pathways associated with stress responses and the control of oxidative stress categories found here [12,27,62]. Thus, these contrasting results suggest the existence of important post-transcriptional regulation in *Coffea*, at least in the genotypes studied here. Other studies have also highlighted that protective metabolites often do not show a clear pattern between transcript accumulation and metabolite/physiological responses in response to stress, being likely to not be transcriptionally regulated. For instance, [63] studied the transcript response to eCO_2_ in *Solanum lycopersicum* and its wild relative *S. pennelli*, and no clear transcriptomic pattern was found, but rather a translational regulatory mechanism, hypothetically involved in the differential ribosomal loading of transcripts in the two species. Additionally, in *Saccharomyces cerevisiae*, relatively few (~15%) of the mRNAs that were translationally up-regulated in response to H_2_O_2_ showed similar increases in transcript levels [64], revealing a complex transcriptional and translational reprogramming to stress. The existence of a complex translational program in *Coffea* would also explain the physiological and biochemical performance of these genotypes [12,26], as well the amplified acclimation responses at the proteomic level [27], namely in Icatu plants, despite the down-regulation of transcripts reported here.

## 4. Materials and Methods

### 4.1. Plant Growth Conditions

Plants of two cropped genotypes from the two main producing coffee species, *Coffea canephora* Pierre ex A. Froehner cv. Conilon Clone 153 (CL153) and *C. arabica* L. cv. Icatu Vermelho (Icatu) were obtained, respectively, from Emcapa and IAC. A total of 26 plants were grown from the seedling stage, for seven years, in 80 L pots in walk-in growth chambers (EHHF 10000, ARALAB, Albarraque, Portugal), under controlled conditions of temperature (25/20 °C, day/night, ±1 °C), irradiance (max. ca. 750 μmol m^−2^ s^−1^ at the upper part of the plant, using a combination of fluorescent, metal halide and halogen lamps to provide a balanced light spectrum), relative humidity (70 ± 2%), photoperiod (12 h), and exposed to ambient (aCO_2_, 380 ± 5 μL L^−1^) or elevated (eCO_2_, 700 ± 5 μL L^−1^) atmospheric [CO_2_] [12]. Plants were maintained without restrictions of nutrients (with fertilization provided as stated in [65]), root growing space, or water (until water deficit experiments), watering the plants every two days to maintain adequate soil moisture.

### 4.2. Imposition and Monitoring of Water Deficit Conditions and Sampling

Plants previously maintained without water restriction were divided into three groups. The first one was maintained under well-watered (WW) conditions, with a leaf predawn water potential (Ψ_pd_) above −0.35 MPa. In the other two groups, drought was imposed by a gradual reduction of irrigation, allowing plants to express their potential acclimation ability for two weeks, to promote Ψ_pd_ decline to values between −1.5 and −2.5 MPa (moderate water deficit—MWD) or below −3.5 MPa (severe water deficit—SWD), representing ca. 80 (WW), 35 (MWD) and 10% (SWD) of maximal water availability in pots [6]. These MWD and SWD conditions were maintained for another two weeks by adding adequate water amounts according to each water deficit level. Samples were then collected for transcriptomic analysis. Exceptionally, Icatu eCO_2_ plants under MWD were submitted to total water withholding in the last 5 days of the 4-week period, to force the reduction of Ψ_pd_, which, even so, did not shift below −0.6 MPa. Leaf Ψ_pd_ was determined immediately after leaf excision, using a pressure chamber (Model 1000, PMS Instrument Co., Albany, OR, USA).

### 4.3. RNA Extraction and Illumina Sequencing

Newly matured leaves from plagiotropic and orthotropic branches from the upper third part (well illuminated) of each plant were collected under photosynthetic steady-state conditions after 2 h of illumination, flash frozen in liquid nitrogen, and stored at −80 °C. Total RNA was extracted from 36 samples (two genotypes × three water treatments × two [CO_2_] × three individual plants) using the Analytik-Jena InnuSPEED Plant RNA Kit (Analytik Jena Innuscreen GmbH, Jena, Germany) following [50]. RNA quantity and quality were determined using a BioDrop Cuvette (BioDrop, UK) and an Agilent 2100 Bioanalyzer (Agilent Technologies, Santa Clara, CA, USA). RNA integrity number (RIN) for the samples ranged from 8.96 to 9.05. The mRNA libraries were constructed with the Illumina TruSeq Stranded mRNA Sample Preparation kit (Illumina, San Diego, CA, USA) and sequenced on an Illumina NovaSeq6000 at Macrogen facilities (Macrogen, Geumcheongu, Seoul, Republic of Korea).

### 4.4. Quality Analysis of Sequencing Data

Raw reads were processed using FastQC version 0.11.9 [66] to remove low-quality reads. FastQ Screen version 0.14 [67] was used to check for contaminants against the genome of the most common model organisms (e.g., *Homo sapiens*, *Mus musculus*, *Rattus norvegicus*, *Drosophila melanogaster*, *Caenorhabditis elegans*, *Saccharomyces cerevisiae*, *Escherichia coli*) and adapter databases (e.g., Mitochondria RNA, PhiX, Vector from UniVec database, FastQ Screen rRNA custom database, and FastQ Screen Adapters database). Since all reads presented an overall good quality, the trimming step was skipped. Recent studies showed that this process is redundant in the quantification of expression data from RNA-seq since most aligners can perform soft-clipping to effectively remove adapter sequences and rescue low-sequencing-quality bases that would be removed by read trimming tools, improving the accuracy in the quantification of gene expression [68].

### 4.5. Reference-Based Mapping and Assembly

The raw reads of Icatu were mapped to the reference genome of *C. arabica* downloaded from the NCBI (https://www.ncbi.nlm.nih.gov/assembly/GCF_003713225.1, accessed on 4 April 2021), while the raw reads of CL153 were mapped to the *C. canephora* genome downloaded from the Coffee Genome Hub (http://coffee-genome.org/download, accessed on 4 April 2021) [69] using STAR version 2.7.8a [70]. Htseq-count v0.11.0 [71] was then used to quantify only uniquely mapped genes. Samtools version 1.12 [72] and gffread version 0.12.1 [73] were used throughout the analysis to obtain general statistics on genome mapping. A principal component analysis (PCA) was performed on the expression data of genes, TMM (trimmed mean of means) normalized and log10-transformed using ggplot2 version 3.3.3 library [74] of R software version 4.0.2 [75]. Through visual inspection of the PCA, replicate 7B was considered an outlier and excluded from downstream analyses (Figure S1).

### 4.6. Identification of Differentially Expressed Genes (DEGs)

DEGs from the plants grown under different water treatments and different [CO_2_] were estimated and compared as follows: MWD-aCO_2_ vs. WWa-CO_2_, SWD-aCO_2_ vs. WW-aCO_2_, MWD-eCO_2_ vs. WW-eCO_2_, and SWD-eCO_2_ vs. WW-eCO_2_. Differential expression analyses were performed using the DEGs commonly found by both DESeq2 version 3.8 [76] and edgeR version 3.26.0 [77]. The resulting values of expression were adjusted using Benjamini and Hochberg’s approach to control the false discovery rate (FDR; [78]). Genes with a normalized non-zero log_2_ fold change (FC) expression and an FDR < 0.01 in both tools were defined as differentially expressed. Python’s matplotlib library was used to plot Venn diagrams and bar plots [79]. Using ggplot2, heatmaps with dendrograms were plotted to visualize DEGs based on the differential expression patterns between the different comparisons. To prevent high DEGs from clustering together without considering their expression pattern, log_2_ FC was scaled by gene expression across treatments (row Z-score).

### 4.7. Drought and eCO_2_ Impact on DEGs Associated with Specific Biochemical Pathways

Due to the fundamental role of photosynthesis, respiration, lipid profile changes, and the antioxidant system in the process of coffee acclimation to environmental stresses, a specific/fine-tuned search was performed among the significant DEGs associated with these processes. According to the reference genome and the UniProtKB database DEGs annotated with the following direct and child GO terms were searched to study their regulation pattern: [Photosynthesis], ‘photosynthesis’ (GO:0015979), ‘photosystem’ (GO:0009521), ‘photosynthetic membrane’ (GO:0034357), ‘photoinhibition’ (GO:0010205), ‘photosynthetic phosphorylation’ (GO:0009777), ‘photosynthetic acclimation’ (GO:0009643), ‘photosynthetic state transition’ (GO:0062055), ‘photosynthetic NADP+ reduction’ (GO:0009780), ‘photosynthetic electron transport chain’ (GO:0009780), ‘photosynthetic electron transport chain’ (GO:0009767), ‘photorespiration’ (GO:0009853), ‘chlorophyll metabolic process’ (GO:0015994), ‘chlorophyll biosynthetic process’ (GO:0015995), and ‘chlorophyll catabolic process’ (GO:0015996); [Cellular respiration], ‘cellular respiration’ (GO:0045333), ‘oxidative phosphorylation’ (GO:0006119), and ‘mitochondrion’ (GO:0005739), [Antioxidant activity], ‘antioxidant activity’ (GO:0016209), and ‘response to oxidative stress’ (GO:0006979); [Lipid metabolism], ‘fatty acid metabolic process’ (GO:0006631), ‘FAD metabolic process’ (GO:0046443), ‘FAD transport’ (GO:0015883), and LOX (GO:0004051, GO:0016702). Additionally, a more specific pathway analysis was conducted where DEGs related to the light reactions of photosynthesis, the Calvin cycle, and photorespiration were visualized through MapMan version 3.6.0RC1 [80].

### 4.8. DEGs Involved in the Response to Water Deprivation and Desiccation

To better understand the impacts of water deficit, a specific search was performed, as described previously, among DEGs annotated with ‘water transport’ (GO:0006833), ‘water homeostasis’ (GO:0030104), ‘response to water’ (GO:0009415), ‘response to water deprivation’ (GO:0009414), and ‘response to desiccation’ (GO:0009269).

### 4.9. Functional Classification of Responsive DEGs

DEGs from CL153 and Icatu comparisons were annotated following the functional annotation of the reference genomes of *C. canephora* and *C. arabica*, respectively, as stated previously. GO enrichment analyses were applied to understand the functional classification of responsive DEGs through another over-representation analysis (ORA) using gProfiler [81] under g:SCS < 0.01. Results were summarized using REVIGO [82] by removing redundant GO terms within a similarity = 0.5. Enrichment non-redundant results were plotted using ggplot2 version 3.3.3 library, using the number of DEGs annotated with each term to set a Counts > 10 cut-off [74]. Since *Coffea* genomic annotations are not complete, namely in terms of KEGG pathways, DEGs were mapped to their *Arabidopsis thaliana* homologs against a local Swissprot database, filtering gene hits by maximum e-value of 1.0 × 10^−3^ and minimum identities of 40% [83], and using blastx from the Basic Local Alignment Search Tool (BLAST) version 2.10.1 command-line tool from the NCBI C++ Toolkit. These annotations were then used to perform an over-representation analysis (ORA) with gProfiler, searching for significantly (g:SCS < 0.01) enriched KEGG pathways.

### 4.10. Quantitative RT-PCR

Twelve transcripts were randomly selected for real-time quantitative PCR (qRT-PCR) to verify the accuracy of the levels of expression obtained under RNA-seq. Genes included: *GMPM1*: 18 kDa seed maturation; *PP2C-51*: protein phosphatase 2C 51-like; *LEA-DC3*: late embryogenesis abundant protein Dc3-like; *DH1a*: dehydrin DH1a; *ATHB22*: homeobox leucine zipper; *SUS2*: sucrose synthase 2-like; *PIP2-2*: aquaporin PIP2-2-like; *XTH6*: xyloglucan endotransglucosylase/hydrolase protein 6; *GOLS2*: galactinol synthase 2-like; *CuSOD1*: Superoxide dismutase [Cu-Zn]; *APX_Chl_*: chloroplast ascorbate peroxidase. All primer sequences are presented in Table S1. The primers were designed using Primer3 web version 4.1.0 [84] with an e-value < 2 × 10^−4^ and a score >41. cDNA was synthesized from 1 μg total RNA using the SensiFASTTM cDNA Synthesis kit (Meridian BioScience, Cincinnati, OH, USA), according to the manufacturer’s recommendations. The presence of a single amplification product of the expected gene size was verified by electrophoresis on a 1.5% agarose gel. PCR reactions were prepared using the SensiFASTTM SYBR No-ROX kit (Meridian BioScience, USA) according to the manufacturer’s protocol. One negative sample was included for each primer pair, in which cDNA was replaced by water. Reactions were carried out in 96-well plates using a qTOWER 2.2 Thermal Cycler (Analytik, Jena, Germany) using the following parameters: hot start activation of the Taq DNA polymerase at 95 °C for 10 min, followed by 40 cycles of denaturation at 95 °C for 15 s, annealing at 60 °C for 30 s, elongation at 72 °C for 30 s. A melting curve analysis was performed at the end of the PCR run by a continuous fluorescence measurement from 55 °C to 95 °C with sequential steps of 0.5 °C for 15 s. A single peak was obtained and no signal was detected in the negative controls. Three technical replicates were used for each analyzed plant. Gene expression was quantified using malate dehydrogenase (*MDH*) and ubiquitin (*UBQ10*) as reference genes [85]. To understand the agreement of these results with the one from RNA-sequencing, heatmaps were constructed considering the levels of transcripts and their expression levels from qRT-PCRs after being log_2_ FC scaled by gene expression across treatments.

## 5. Conclusions

In this study, we showed how the single and combined effects of drought and eCO_2_ triggered wide but differential responses at the transcriptional level in *Coffea*.

MWD had a minor impact on the number of transcripts differentially regulated by Icatu and CL153, contrary to SWD where a high number of DEGs were reported, being mostly down-regulated. eCO_2_ attenuated the impacts of drought in the two genotypes, but especially in Icatu, in agreement with the contrasting physiological tolerance previously reported in these genotypes.

There was a predominance of protective and ROS-scavenging genes, directly or indirectly related to ABA signaling pathways involving *Coffea* tolerance responses. These genes were also involved in water deprivation and desiccation processes, such as *LEA* and protein phosphatases in Icatu and Aspartic Protease in Guard Cell 1-like and dehydrins in CL153, being their expression confirmed by qRT-PCR.

Enrichment analysis of GO and KEGG pathways revealed different regulatory mechanisms of Icatu and CL153 in response to drought, agreeing with the minor effects of MWD and the positive action of eCO_2_. However, a clear effect on photosynthetic pathways was recorded, namely under SWD and eCO_2_, contrary to previous physiological and biochemical studies.

The existence of a complex post-transcriptional regulatory mechanism is suggested to occur in *Coffea* explaining the discrepancies between transcriptional vs. proteomic and physiological data in these genotypes.

## Figures and Tables

**Table 1:** Regulation patterns of the top 20 up- and down-differentially expressed genes (DEGs) related to drought responses in *Coffea arabica* cv. Icatu (Icatu) plants grown under MWD or SWD in comparison with WW plants grown under aCO_2_ or eCO_2_, at 25/20 °C (day/night). Blue: down-regulated DEGs; red: up-regulated DEGs.

Gene ID	Protein Name	aCO_2_		eCO_2_	
		MWD	SWD	MWD	SWD
LOC113741996	Late Embryogenesis Abundant protein Dc3-like		5.51		7.19
LOC113704200	Homeobox-leucine zipper protein ATHB-12-like	4.50	4.83		6.75
LOC113740436	Late Embryogenesis Abundant protein Dc3-like		3.58		6.10
LOC113727829	Galactinol synthase 2-like	2.99	4.29		6.07
LOC113706564	Protein phosphatase 2C 51-like		4.43		6.06
LOC113733000	Galactinol synthase 2-like		4.89	3.45	5.79
LOC113703008	Protein phosphatase 2C 51-like		5.62		5.54
LOC113727830	Galactinol synthase 2-like		4.91		5.43
LOC113743599	NAC domain-containing protein 72-like		3.84		5.41
LOC113689826	NAC domain-containing protein 72-like		3.83		4.86
LOC113740563	HVA22-like protein e	4.25	3.96		4.65
LOC113695700	Protein LE25-like		3.46		4.65
LOC113692189	Late Embryogenesis Abundant protein 46-like				4.64
LOC113740410	HVA22-like protein e	4.94	4.7		4.61
LOC113692190	18 kDa seed maturation protein-like				4.53
LOC113729974	Acidic endochitinase SE2-like				4.15
LOC113740137	Protein Aspartic Protease in Guard Cell 1-like				4.05
LOC113729880	Homeobox-leucine zipper protein ATHB-12-like	3.79	3.72		3.63
LOC113733488	Late Embryogenesis Abundant protein-like				3.62
LOC113726525	Zinc finger protein ZAT10-like		4.46		3.54
LOC113709223	Aquaporin TIP1-3-like	−4.55	−4.35		−3.52
LOC113706149	Probable LRR receptor-like serine/threonine-protein kinase At1g34110				−4.01
LOC113702113	Serine/threonine-protein kinase SAPK1-like isoform X3		−3.15		−4.26
LOC113742299	Protein Aspartic Protease in Guard Cell 1-like	−4.16	−7.11		−4.32
LOC113733193	Probable aquaporin PIP1-2		−4.03		
LOC113716691	Protein Aspartic Protease in Guard Cell 1-like	−2.96	−4.37		
LOC113695827	Pathogenesis-related protein PR-1-like		−4.83		
LOC113742753	Aquaporin TIP2-1	−3.25	−5.97		−4.38
LOC113741887	Ethylene-responsive transcription factor WIN1-like				−4.46
LOC113707187	Aquaporin TIP4-1-like		−5.45		−4.93
LOC113740136	Protein Aspartic Protease in Guard Cell 1-like	−3.8	−6.26		−4.93
LOC113732068	Protein Eceriferum 1-like		−4.20		−5.03
LOC113735055	Basic endochitinase-like	−4.17	−7.54		−5.58
LOC113742697	Protein Aspartic Protease in Guard Cell 1-like	−3.81	−6.63		−5.62
LOC113716416	Acidic endochitinase-like		−4.09		−5.94
LOC113701593	PLAT domain-containing protein 3-like	−7.32	−8.00		−6.22
LOC113742441	Protein Aspartic Protease in Guard Cell 1-like				−8.98
LOC113737208	Basic endochitinase-like	−4.41	−7.12		
LOC113739398	Ethylene-responsive transcription factor WIN1-like		−7.71		
LOC113697821	Probable xyloglucan endotransglucosylase/hydrolase protein 6	−4.72	−12.31		−11.17

**Table 2:** Regulation patterns of differentially expressed genes (DEGs) related to drought responses in *Coffea canephora* cv. CL153 (CL153) grown under MWD or SWD in comparison with WW plants grown under ambient aCO_2_ or eCO_2_, at 25/20 °C (day/night). Blue: down-regulated DEGs; red: up-regulated DEGs.

Gene ID	Protein Name	aCO_2_		eCO_2_	
		MWD	SWD	MWD	SWD
Cc07_g07560	Probable xyloglucan endotransglucosylase/hydrolase protein 6	−6.90	−5.85		
Cc04_g09640	Protein Aspartic Protease in Guard Cell 1-like	−5.03	−4.92		
Cc04_g07360	Putative protein Aspartic Protease in Guard Cell 1-like	4.89	4.07		3.96
Cc04_g07380	Putative protein Aspartic Protease in Guard Cell 1-like	4.27	3.17		2.80
Cc07_g10030	Dehydrin DH1a	6.05	5.47		2.37
Cc01_g11790	Mitogen-activated protein kinase 3				1.45
Cc06_g09540	Multiprotein-bridging factor 1c				2.34
Cc04_g08280	Putative movement protein binding protein 2C		−2.86		−1.92
Cc06_g15980	18 kDa seed maturation protein	9.18	8.79		2.92
Cc04_g07350	Putative protein Aspartic Protease in Guard Cell 1-like		−5.86		
Cc04_g07330	Putative protein Aspartic Protease in Guard Cell 1-like	−7.93	−8.16		
Cc02_g17270	Putative Late Embryogenesis Abundant protein	4.32	3.58		
Cc01_g08980	Late Embryogenesis Abundant protein hydroxyproline-rich glycoprotein family		−3.25		
Cc04_g07370	Putative protein Aspartic Protease in Guard Cell 1-like		−3.48		
Cc02_g15480	Cellulose synthase A catalytic subunit 8 [UDP-forming]		−5.28		
Cc07_g15660	C2 domain-containing protein		−1.73		

## Data Availability

Raw reads have been deposited in the NCBI Sequence Read Archive, BioProject accession PRJNA787748.

## Supplementary Materials

The following supporting information can be downloaded at: https://www.mdpi.com/article/10.3390/ijms24043210/s1.

## References

[1] Solomon S., Plattner G.K., Knutti R., Friedlingstein P. (2009). Irreversible climate change due to carbon dioxide emissions. Proc. Natl. Acad. Sci. USA.

[2] Raza A., Mubarik M.S., Sharif R., Habib M., Jabeen W., Zhang C., Chen H., Chen Z.-H., Siddique K.H.M., Zhuang W. (2022). The Plant Genome Developing drought-smart, ready-to-grow future crops. Plant Genome.

[3] Brodribb T.J., McAdam S.A.M. (2017). Evolution of the Stomatal Regulation of Plant Water Content. Plant Physiol..

[4] Chaves M.M., Maroco J.P., Pereira J.S. (2003). Understanding plant responses to drought-From genes to the whole plant. Funct. Plant Biol..

[5] Muller B., Pantin F., Génard M., Turc O., Freixes S., Piques M., Gibon Y. (2011). Water deficits uncouple growth from photosynthesis, increase C content, and modify the relationships between C and growth in sink organs. J. Exp. Bot..

[6] Ramalho J.C., DaMatta F.M., Rodrigues A.P., Scotti-Campos P., Pais I., Batista-Santos P., Partelli F.L., Ribeiro A., Lidon F.C., Leitão A.E. (2014). Cold impact and acclimation response of *Coffea* spp. plants. Theor. Exp. Plant Physiol..

[7] Ramalho J.C., Rodrigues A.P., Lidon F.C., Marques L.M.C., Leitão A.E., Fortunato A.S., Pais I.P., Silva M.J., Scotti-Campos P., Lopes A. (2018). Stress cross-response of the antioxidative system promoted by superimposed drought and cold conditions in *Coffea* spp.. PLoS ONE.

[8] Fahad S., Bajwa A.A., Nazir U., Anjum S.A., Farooq A., Zohaib A., Sadia S., Nasim W., Adkins S., Saud S. (2017). Crop production under drought and heat stress: Plant responses and management options. Front. Plant Sci..

[9] Chaves M.M., Flexas J., Pinheiro C. (2009). Photosynthesis under drought and salt stress: Regulation mechanisms from whole plant to cell. Ann. Bot..

[10] Ainsworth E.A., Rogers A. (2007). The response of photosynthesis and stomatal conductance to rising [CO_2_]: Mechanisms and environmental interactions. Plant Cell Environ..

[11] Leakey A.D.B., Ainsworth E.A., Bernacchi C.J., Rogers A., Long S.P., Ort D.R. (2009). Elevated CO_2_ effects on plant carbon, nitrogen, and water relations: Six important lessons from FACE. J. Exp. Bot..

[12] Semedo J.N., Rodrigues A.P., Lidon F.C., Pais I.P., Marques I., Gouveia D., Armengaud J., Silva M.J., Martins S., Semedo M.C. (2021). Intrinsic non-stomatal resilience to drought of the photosynthetic apparatus in *Coffea* spp. is strengthened by elevated air [CO_2_]. Tree Physiol..

[13] Tausz-Posch S., Tausz M., Bourgault M. (2020). Elevated [CO_2_] effects on crops: Advances in understanding acclimation, nitrogen dynamics and interactions with drought and other organisms. Plant Biol..

[14] De Souza A.P., Cocuron J.-C., Garcia A.C., Alonso A.P., Buckeridge M.S. (2015). Changes in Whole-Plant Metabolism during the Grain-Filling Stage in Sorghum Grown under Elevated CO_2_ and Drought. Plant Physiol..

[15] Gray S.B., Dermody O., Klein S.P., Locke A.M., McGrath J.M., Paul R.E., Rosenthal D.M., Ruiz-Vera U.M., Siebers M.H., Strellner R. (2016). Intensifying drought eliminates the expected benefits of elevated carbon dioxide for soybean. Nat. Plants.

[16] ICO International Coffee Organization-What’s New. https://www.ico.org/.

[17] DaMatta F.M., Rahn E., Läderach P., Ghini R., Ramalho J.C. (2019). Why could the coffee crop endure climate change and global warming to a greater extent than previously estimated?. Clim. Change.

[18] Cassamo C.T., Mangueze A.V.J., Leitão A.E., Pais I.P., Moreira R., Campa C., Chiulele R., Reis F.O., Marques I., Scotti-Campos P. (2022). Shade and Altitude Implications on the Physical and Chemical Attributes of Green Coffee Beans from Gorongosa Mountain, Mozambique. Agronomy.

[19] DaMatta F.M., Cochicho Ramalho J.D. (2006). Impacts of drought and temperature stress on coffee physiology and production: A review. Braz. J. Plant Physiol..

[20] Vinecky F., Davrieux F., Mera A.C., Alves G.S.C., Lavagnini G., Leroy T., Bonnot F., Rocha O.C., Bartholo G.F., Guerra A.F. (2017). Controlled irrigation and nitrogen, phosphorous and potassium fertilization affect the biochemical composition and quality of Arabica coffee beans. J. Agric. Sci..

[21] Avila R.T., Cardoso A.A., de Almeida W.L., Costa L.C., Machado K.L.G., Barbosa M.L., de Souza R.P.B., Oliveira L.A., Batista D.S., Martins S.C.V. (2020). Coffee plants respond to drought and elevated [CO_2_] through changes in stomatal function, plant hydraulic conductance, and aquaporin expression. Environ. Exp. Bot..

[22] Avila R.T., de Almeida W.L., Costa L.C., Machado K.L.G., Barbosa M.L., de Souza R.P.B., Martino P.B., Juárez M.A.T., Marçal D.M.S., Martins S.C.V. (2020). Elevated air [CO_2_] improves photosynthetic performance and alters biomass accumulation and partitioning in drought-stressed coffee plants. Environ. Exp. Bot..

[23] Sanches R.F.E., da Cruz Centeno D., Braga M.R., da Silva E.A. (2020). Impact of high atmospheric CO_2_ concentrations on the seasonality of water-related processes, gas exchange, and carbohydrate metabolism in coffee trees under field conditions. Clim. Chang..

[24] Catarino I.C.A., Monteiro G.B., Ferreira M.J.P., Torres L.M.B., Domingues D.S., Centeno D.C., Lobo A.K.M., Silva E.A. (2021). Elevated [CO_2_] Mitigates Drought Effects and Increases Leaf 5-O-Caffeoylquinic Acid and Caffeine Concentrations during the Early Growth of Coffea Arabica Plants. Front. Sustain. Food Syst..

[25] Rodrigues A.M., Jorge T., Osorio S., Pott D.M., Lidon F.C., DaMatta F.M., Marques I., Ribeiro-barros A.I., Ramalho J.C., António C. (2021). Primary Metabolite Profile Changes in *Coffea* spp. Promoted by Single and Combined Exposure to Drought and Elevated CO_2_ Concentration. Metabolites.

[26] Dubberstein D., Lidon F.C., Rodrigues A.P., Semedo J.N., Marques I., Rodrigues W.P., Gouveia D., Armengaud J., Semedo M.C., Martins S. (2020). Resilient and Sensitive Key Points of the Photosynthetic Machinery of *Coffea* spp. to the Single and Superimposed Exposure to Severe Drought and Heat Stresses. Front. Plant Sci..

[27] Marques I., Rodrigues A.P., Gouveia D., Lidon F.C., Martins S., Semedo M.C., Gaillard J.C., Pais I.P., Semedo J.N., Scotti-Campos P. (2022). High-resolution shotgun proteomics reveals that increased air [CO_2_] amplifies the acclimation response of *Coffea* species to drought regarding antioxidative, energy, sugar, and lipid dynamics. J. Plant Physiol..

[28] Vranová E., Langebartels C., Van Montagu M., Inzé D., Van Camp W. (2000). Oxidative stress, heat shock and drought differentially affect expression of a tobacco protein phosphatase 2C. J. Exp. Bot..

[29] Merlot S., Gosti F., Guerrier D., Vavasseur A., Giraudat J. (2001). The ABI1 and ABI2 protein phosphatases 2C act in a negative feedback regulatory loop of the abscisic acid signalling pathway. Plant J..

[30] Komatsu K., Suzuki N., Kuwamura M., Nishikawa Y., Nakatani M., Ohtawa H., Takezawa D., Seki M., Tanaka M., Taji T. (2013). Group A PP2Cs evolved in land plants as key regulators of intrinsic desiccation tolerance. Nat. Commun..

[31] Sun L., Wang Y.P., Chen P., Ren J., Ji K., Li Q., Li P., Dai S.J., Leng P. (2011). Transcriptional regulation of SlPYL, SlPP2C, and SlSnRK2 gene families encoding ABA signal core components during tomato fruit development and drought stress. J. Exp. Bot..

[32] Chen J., Zhang D., Zhang C., Xia X., Yin W., Tian Q. (2015). A Putative PP2C-encoding gene negatively regulates ABA signaling in populus euphratica. PLoS ONE.

[33] Zhang F., Fu X., Lv Z., Shen Q., Yan T., Jiang W., Wang G., Sun X., Tang K. (2014). Type 2C phosphatase 1 of *Artemisia annua* L. is a negative regulator of ABA signaling. Biomed Res. Int..

[34] D’Ippólito S., Rey-Burusco M.F., Feingold S.E., Guevara M.G. (2021). Role of proteases in the response of plants to drought. Plant Physiol. Biochem..

[35] Meinhard M., Rodriguez P.L., Grill E. (2002). The sensitivity of ABI2 to hydrogen peroxide links the abscisic acid-response regulator to redox signalling. Planta.

[36] Mittler R. (2002). Oxidative stress, antioxidants and stress tolerance. Trends Plant Sci..

[37] Liu Y., Song Q., Li D., Yang X., Li D. (2017). Multifunctional roles of plant dehydrins in response to environmental stresses. Front. Plant Sci..

[38] Santos A.B., Mazzafera P. (2012). Dehydrins Are Highly Expressed in Water-Stressed Plants of Two Coffee Species. Trop. Plant Biol..

[39] Fernandes I., Marques I., Paulo O.S., Batista D., Partelli F.L., Lidon F.C., DaMatta F.M., Ramalho J.C., Ribeiro-Barros A.I. (2021). Understanding the impact of drought in *Coffea* genotypes: Transcriptomic analysis supports a common high resilience to moderate water deficit but a genotype dependent sensitivity to severe water deficit. Agronomy.

[40] Noctor G., Reichheld J.P., Foyer C.H. (2018). ROS-related redox regulation and signaling in plants. Semin. Cell Dev. Biol..

[41] Dumanović J., Nepovimova E., Natić M., Kuča K., Jaćević V. (2021). The Significance of Reactive Oxygen Species and Antioxidant Defense System in Plants: A Concise Overview. Front. Plant Sci..

[42] Martins M.Q., Rodrigues W.P., Fortunato A.S., Leitão A.E., Rodrigues A.P., Pais I.P., Martins L.D., Silva M.J., Reboredo F.H., Partelli F.L. (2016). Protective response mechanisms to heat stress in interaction with high [CO_2_] conditions in *Coffea* spp.. Front. Plant Sci..

[43] Rodrigues W.P., Martins M.Q., Fortunato A.S., Rodrigues A.P., Semedo J.N., Simões-Costa M.C., Pais I.P., Leitão A.E., Colwell F., Goulao L. (2016). Long-term elevated air [CO_2_] strengthens photosynthetic functioning and mitigates the impact of supra-optimal temperatures in tropical *Coffea arabica* and *C. canephora* species. Glob. Chang. Biol..

[44] Vinci G., Marques I., Rodrigues A.P., Martins S., Leitão A.E., Semedo M.C., Silva M.J., Lidon F.C., DaMatta F.M., Ribeiro-Barros A.I. (2022). Protective Responses at the Biochemical and Molecular Level Differ between a *Coffea arabica* L. Hybrid and Its Parental Genotypes to Supra-Optimal Temperatures and Elevated Air [CO_2_]. Plants.

[45] Fortunato A.S., Lidon F.C., Batista-Santos P., Eduardo Leitão A., Pais I.P., Ribeiro A.I., Cochicho Ramalho J. (2010). Biochemical and molecular characterization of the antioxidative system of *Coffea* sp. under cold conditions in genotypes with contrasting tolerance. J. Plant Physiol..

[46] Ramalho J.C., Fortunato A.S., Goulao L.F., Lidon F.C. (2013). Cold-induced changes in mineral content in leaves of *Coffea* spp. Identification of descriptors for tolerance assessment. Biol. Plant..

[47] Ramalho J.C., Campos P.S., Teixeira M., Nunes M.A. (1998). Nitrogen dependent changes in antioxidant system and in fatty acid composition of chloroplast membranes from *Coffea arabica* L. plants submitted to high irradiance. Plant Sci..

[48] Ramalho J.C., Pons T.L., Groeneveld H.W., Nunes M.A. (1997). Photosynthetic responses of *Coffea arabica* leaves to a short-term high light exposure in relation to N availability. Physiol. Plant..

[49] Miniussi M., Del Terra L., Savi T., Pallavicini A., Nardini A. (2015). Aquaporins in Coffea arabica L.: Identification, expression, and impacts on plant water relations and hydraulics. Plant Physiol. Biochem..

[50] Marques I., Fernandes I., Paulo O.S., Lidon F.C., DaMatta F.M., Ramalho J.C., Ribeiro-barros A.I. (2021). A transcriptomic approach to understanding the combined impacts of supra-optimal temperatures and CO_2_ revealed different responses in the polyploid *Coffea arabica* and its diploid progenitor *C. canephora*. Int. J. Mol. Sci..

[51] Devnarain N., Crampton B.G., Olivier N., van der Westhuyzen C., Becker J.V.W., O’Kennedy M.M. (2019). Transcriptomic analysis of a *Sorghum bicolor* landrace identifies a role for beta-alanine betaine biosynthesis in drought tolerance. S. Afr. J. Bot..

[52] Aziz M.A., Sabeem M., Mullath S.K., Brini F., Masmoudi K. (2021). Plant Group II LEA Proteins: Intrinsically Disordered Structure for Multiple Functions in Response to Environmental Stresses. Biomolecules.

[53] Magwanga R.O., Lu P., Kirungu J.N., Lu H., Wang X., Cai X., Zhou Z., Zhang Z., Salih H., Wang K. (2018). Characterization of the late embryogenesis abundant (LEA) proteins family and their role in drought stress tolerance in upland cotton. BMC Genet..

[54] Buckley T.N. (2019). How do stomata respond to water status?. New Phytol..

[55] Cui L., Yao S., Dai X., Yin Q., Liu Y., Jiang X., Wu Y., Qian Y., Pang Y., Gao L. (2016). Identification of UDP-glycosyltransferases involved in the biosynthesis of astringent taste compounds in tea (*Camellia sinensis*). J. Exp. Bot..

[56] Dong N.-Q., Sun Y., Guo T., Shi C.-L., Zhang Y.-M., Kan Y., Xiang Y.-H., Zhang H., Yang Y.-B., Li Y.-C. (2020). UDP-glucosyltransferase regulates grain size and abiotic stress tolerance associated with metabolic flux redirection in rice. Nat. Commun..

[57] Jarzyniak K.M., Jasiński M. (2014). Membrane transporters and drought resistance-A complex issue. Front. Plant Sci..

[58] Ahmed U., Rao M.J., Qi C., Xie Q., Noushahi H.A., Yaseen M., Shi X., Zheng B. (2021). Expression profiling of flavonoid biosynthesis genes and secondary metabolites accumulation in populus under drought stress. Molecules.

[59] Watkins J.M., Hechler P.J., Muday G.K. (2014). Ethylene-induced flavonol accumulation in guard cells suppresses reactive oxygen species and moderates stomatal aperture. Plant Physiol..

[60] Takahama U. (2004). Oxidation of vacuolar and apoplastic phenolic substrates by peroxidase: Physiological significance of the oxidation reactions. Phytochem. Rev..

[61] Ferreres F., Figueiredo R., Bettencourt S., Carqueijeiro I., Oliveira J., Gil-Izquierdo A., Pereira D.M., Valentão P., Andrade P.B., Duarte P. (2011). Identification of phenolic compounds in isolated vacuoles of the medicinal plant *Catharanthus roseus* and their interaction with vacuolar class III peroxidase: An H_2_O_2_ affair?. J. Exp. Bot..

[62] Marques I., Gouveia D., Gaillard J.C., Martins S., Semedo M.C., Lidon F.C., DaMatta F.M., Ribeiro-Barros A.I., Armengaud J., Ramalho J.C. (2022). Next-Generation Proteomics Reveals a Greater Antioxidative Response to Drought in *Coffea arabica* than in *Coffea Canephora*. Agronomy.

[63] Gray S.B., Rodriguez-Medina J., Rusoff S., Toal T.W., Kajala K., Runcie D.E., Brady S.M. (2020). Translational regulation contributes to the elevated CO_2_ response in two *Solanum* species. Plant J..

[64] Shenton D., Smirnova J.B., Selley J.N., Carroll K., Hubbard S.J., Pavitt G.D., Ashe M.P., Grant C.M. (2006). Global Translational Responses to Oxidative Stress Impact upon Multiple Levels of Protein Synthesis. J. Biol. Chem..

[65] Ramalho J.C., Rodrigues A.P., Semedo J.N., Pais I.P., Martins L.D., Simões-Costa M.C., Leitão A.E., Fortunato A.S., Batista-Santos P., Palos I.M. (2013). Sustained photosynthetic performance of *Coffea* spp. under long-term enhanced [CO_2_]. PLoS ONE.

[66] Andrews S. (2010). FastQC: A Quality Control Tool for High Throughput Sequence Data. http://www.bioinformatics.babraham.ac.uk/projects/fastqc.

[67] Wingett S.W., Andrews S. (2018). FastQ Screen: A tool for multi-genome mapping and quality control. F1000Research.

[68] Liao Y., Shi W. (2020). Read trimming is not required for mapping and quantification of RNA-seq reads at the gene level. NAR Genom. Bioinforma..

[69] Denoeud F., Carretero-Paulet L., Dereeper A., Droc G., Guyot R., Pietrella M., Zheng C., Alberti A., Anthony F., Aprea G. (2014). The coffee genome provides insight into the convergent evolution of caffeine biosynthesis. Science.

[70] Dobin A., Davis C.A., Schlesinger F., Drenkow J., Zaleski C., Jha S., Batut P., Chaisson M., Gingeras T.R. (2013). STAR: Ultrafast universal RNA-seq aligner. Bioinformatics.

[71] Anders S., Pyl P.T., Huber W. (2015). HTSeq—A Python framework to work with high-throughput sequencing data. Bioinformatics.

[72] Li H., Handsaker B., Wysoker A., Fennell T., Ruan J., Homer N., Marth G., Abecasis G., Durbin R. (2009). The Sequence Alignment/Map format and SAMtools. Bioinformatics.

[73] Pertea G.G. (2015). gffread: GFF/GTF Utility Providing Format Conversions, Region Filtering, FASTA Sequence Extraction and More. https://github.com/gpertea/gffread.

[74] Wickham H. (2016). ggplot2: Elegant Graphics for Data Analysis.

[75] Team R.C. (2018). R: A Language and Environment for Statistical Computing.

[76] Love M.I., Huber W., Anders S. (2014). Moderated estimation of fold change and dispersion for RNA-seq data with DESeq2. Genome Biol..

[77] Robinson M.D., McCarthy D.J., Smyth G.K. (2009). edgeR: A Bioconductor package for differential expression analysis of digital gene expression data. Bioinformatics.

[78] Benjamini Y., Hochberg Y. (2016). On the Adaptive Control of the False Discovery Rate in Multiple Testing With Independent Statistics. J. Educ. Behav. Stat..

[79] Hunter J.D. (2007). Matplotlib: A 2D graphics environment. Comput. Sci. Eng..

[80] Thimm O., Bla È Sing O., Gibon Y., Nagel A., Meyer S., Kru È Ger P., Selbig J., Mu È Ller L.A., Rhee S.Y., Stitt M. (2004). MAPMAN: A user-driven tool to display genomics data sets onto diagrams of metabolic pathways and other biological processes. Plant J..

[81] Raudvere U., Kolberg L., Kuzmin I., Arak T., Adler P., Peterson H., Vilo J. (2019). G: Profiler: A web server for functional enrichment analysis and conversions of gene lists (2019 update). Nucleic Acids Res..

[82] Supek F., Bošnjak M., Škunca N., Šmuc T. (2011). Revigo summarizes and visualizes long lists of gene ontology terms. PLoS ONE.

[83] Chen C., Huang H., Wu C.H. (2017). Protein bioinformatics databases and resources. Methods in Molecular Biology.

[84] Untergasser A., Cutcutache I., Koressaar T., Ye J., Faircloth B.C., Remm M., Rozen S.G. (2012). Primer3-new capabilities and interfaces. Nucleic Acids Res..

[85] Martins M.Q., Fortunato A.S., Rodrigues W.P., Partelli F.L., Campostrini E., Lidon F.C., DaMatta F.M., Ramalho J.C., Ribeiro-Barros A.I. (2017). Selection and Validation of Reference Genes for Accurate RT-qPCR Data Normalization in *Coffea* spp. under a Climate Changes Context of Interacting Elevated [CO_2_] and Temperature. Front. Plant Sci..

