# Supplementary material for: Overexpression of Water-Responsive Genes Promoted by Elevated CO2 Reduces ROS and Enhances Drought Tolerance in Coffea Species
Source: Int J Mol Sci. 2023 Feb 6;24(4):3210. doi: 10.3390/ijms24043210 (PMC9966387; doi:10.3390/ijms24043210)
Supplement: Supplementary file 1 [file ijms-24-03210-s001.zip › ijms-2086788-supplementary.pdf]

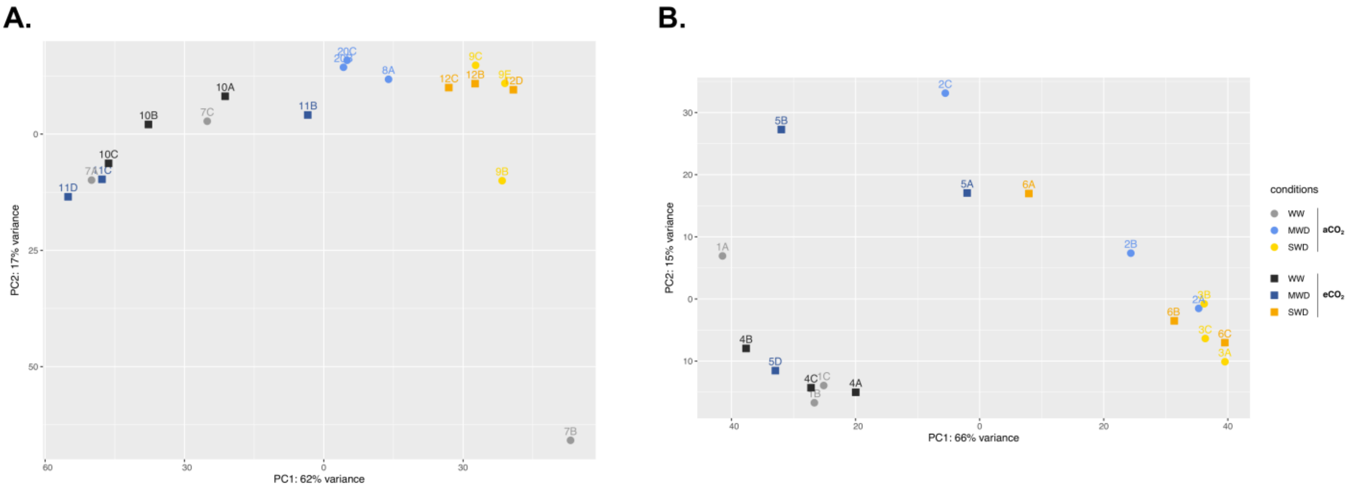

Figure S1. Principal component analysis (PCA) of TMM normalized and rlog transformed gene expression data from (**A**) *Coffea arabica* cv. Icatu (Icatu) and (**B**) *C. canephora* cv. CL153 (CL153). Plants were grown in three different water conditions, well-watered (WW), moderate water deficit (MWD), and severe water deficit (SWD), under either ambient air 380 μL L^{-1} [CO_2] (aCO_2) or elevated 700 μL L^{-1} [CO_2] (eCO_2) at 25/20 °C (day/night). Letters correspond to biological replications. Percentages of variance are indicated on each axis.

A.

B.

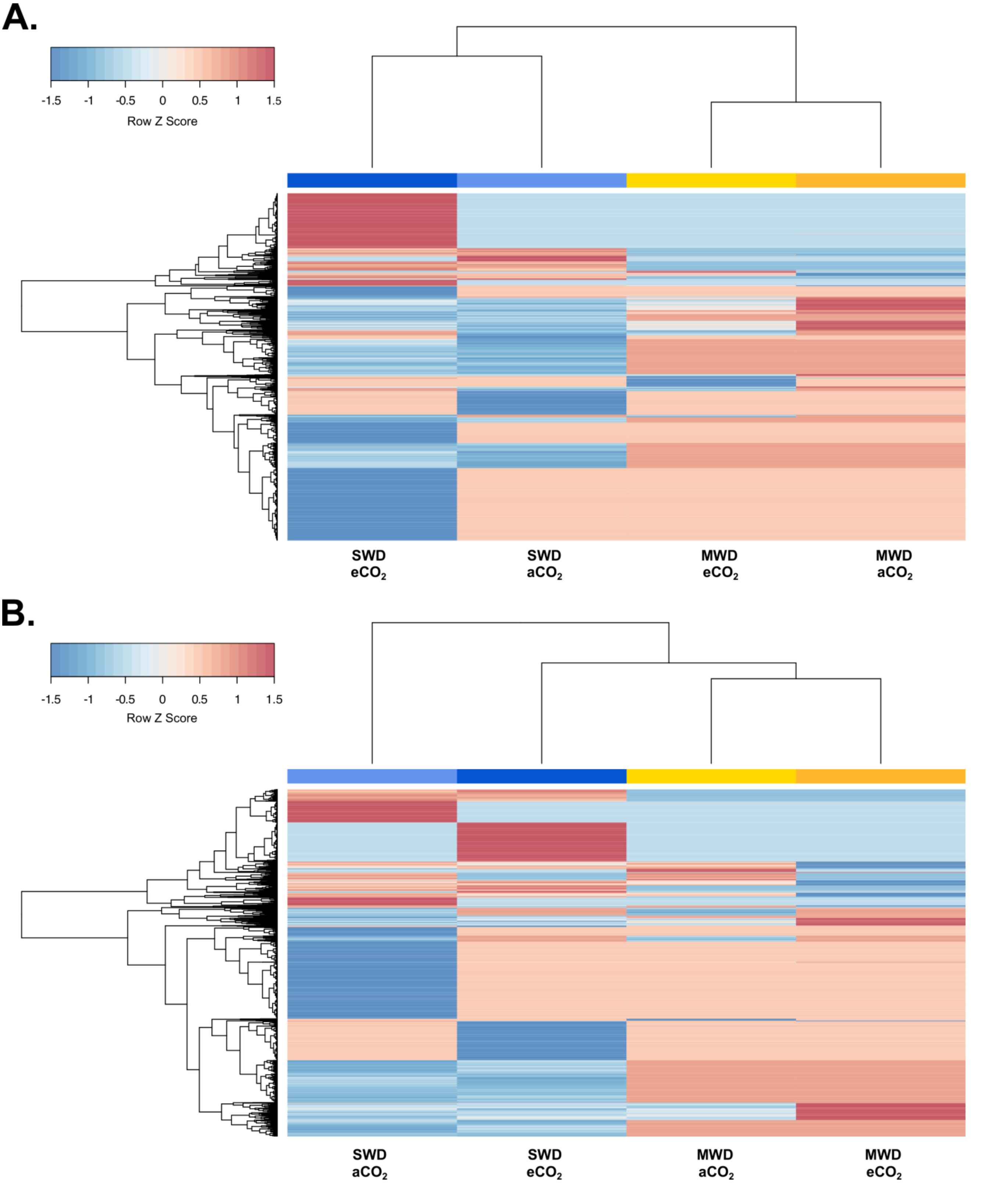

Figure S2. Clustered heatmaps and dendrograms of the normalized log2 fold change (FC) visualizing the expression of all significant (FDR < 0.01) differentially expressed genes (DEGs) in (**A**) Icatu and (**B**) CL153 plants grown under moderate water deficit (MWD) or severe water deficit (SWD) in comparison with well-watered plants, under either ambient air 380 μL L^{-1} [CO_2] (aCO_2) or elevated 700 μL L^{-1} [CO_2] (eCO_2), at 25/20 °C (day/night). Values were scaled by row using Z-scores. Hot colors represent up-regulated DEGs and cold colors represent down-regulated DEGs. Column

colors group comparisons by water treatments (yellow: MWD; blue: SWD) (light colors represent aCO_2, while dark colors represent eCO_2).

Table S1. Primers used in this study for qRT-PCR. ASPG1: Aspartic Protease in Guard Cell 1; GMPM1: 18 kDa seed maturation; PP2C51: protein phosphatase 2C 51-like; LEADC3: late embryogenesis abundant protein Dc3-like; DH1a: dehydrin DH1a; ATHB22: homeobox leucine zipper; SUS2: sucrose synthase 2-like; PIP2: aquaporin PIP2-2-like; XTH6: xyloglucan endotransglucosylase/hydrolase protein 6; GOLS2: galactinol synthase 2-like; CuSOD1: Superoxide dismutase [Cu-Zn]; APXChl: chloroplast ascorbate peroxidase.

Table S2. Summary of sequencing and mapping of reads from *Coffea arabica* cv. Icatu (Icatu) and *C. canephora* cv. CL153 (CL153) grown under moderate water deficit (MWD) or severe water deficit (SWD), and control well-watered plants, either under ambient 380 μL L^{-1} [CO_2] (aCO_2) or elevated 700 μL L^{-1} [CO_2] (eCO_2) at 25/20 °C (day/night). Raw reads: number of reads obtained after sequencing. Uniquely mapped reads: number of reads aligned to a unique position. % Unique: proportion of reads aligned to a unique position compared to the number of raw reads. Multiple mapped reads: number of reads aligned to exons of several overlapping genes. % Multiple: proportion of reads aligned to exons of several overlapping genes compared to the number of raw reads. Unmapped reads: number of non-aligning reads. % Unmapped: proportion of non-aligning reads compared to the number of raw reads.

Table S3. Number of total expressed genes in *Coffea arabica* cv. Icatu (Icatu) and *C. canephora* cv. CL153 (CL153), number of differentially expressed genes (DEGs) detected by DESeq2 and edgeR, with log2 fold-change (FC) ≠ 0 and false discovery rate (FDR) < 0.01, and number of overlapping DEGs from both analyses (% DEGs relative to the average of genes expressed by treated and control plants). Plants were grown in three different water conditions, well-watered (control), moderate water deficit (MWD), and severe water deficit (SWD), under either ambient air 380 μL L^{-1} [CO_2] (aCO_2) or elevated 700 μL L^{-1} [CO_2] (eCO_2), at 25/20 °C (day/night). DEGs represent the number of significant genes found to be differently expressed between each stress water treatment and the control (respectively, MWD vs. WW and SWD vs. WW) [overlap % is the proportion of DEGs detected by both edgeR and DESeq2 related to the average of genes expressed by control and treated plants].

Table S4. Lists of up- and down-regulated differentially expressed genes (DEGs) found in *Coffea arabica* cv. Icatu (Icatu) plants grown under moderate water deficit (MWD) or severe water deficit (SWD) in comparison with well-watered (WW) conditions, under ambient 380 μL L^{-1} [CO_2] (aCO_2) or elevated 700 μL L^{-1} [CO_2] (eCO_2), at 25/20 °C (day/night). Gene identification and protein name according to *Coffea arabica* functional annotation retrieved from NCBI (https://www.ncbi.nlm.nih.gov/assembly/GCF_003713225.1, accessed on 4 April 2021). DEGs were selected with both DESeq2 and edgeR and filtered by log2 fold-change (FC) ≠ 0 and false discovery rate (FDR) < 0.01.

Table S5. Lists of up- and down-regulated differentially expressed genes (DEGs) found in *Coffea canephora* cv. CL153 (CL153) plants grown under moderate water deficit (MWD) or severe water deficit (SWD) in comparison with well-watered (WW) conditions, under ambient 380 μL L^{-1} [CO_2] (aCO_2) or elevated 700 μL L^{-1} [CO_2] (eCO_2), at 25/20 °C (day/night). Gene identification and protein name according to *Coffea canephora* functional annotation retrieved from the Coffee Genome Hub (http://coffee-genome.org, accessed on 4 April 2021). DEGs were selected with both DESeq2 and edgeR and filtered by log2 fold-change (FC) ≠ 0 and false discovery rate (FDR) < 0.01.

Table S6. Regulation patterns of biochemical-related differentially expressed genes (DEGs) found in plants of *Coffea arabica* cv. Icatu (Icatu) grown under moderate water deficit (MWD) or severe water deficit (SWD) in comparison with well-watered (WW) plants, under ambient air 380 μL L^{-1} [CO_2] (aCO_2) or elevated 700 μL L^{-1} [CO_2] (eCO_2), at 25/20 °C (day/night). Red represents up-regulated DEGs and blue represents down-regulated DEGs.

Table S7. Regulations pattern of biochemical-related differentially expressed genes (DEGs) found in plants of *Coffea canephora* cv. CL153 (CL153) grown under moderate water deficit (MWD) or severe water deficit (SWD) in comparison with well-watered (WW) plants, under ambient air 380 μL

L^{-1} [CO_2] (aCO_2) or elevated 700 μL L^{-1} [CO_2] (eCO_2), at 25/20 °C (day/night). Red represents up-regulated DEGs and blue represents down-regulated DEGs.

Table S8. Regulation patterns of *Coffea arabica* cv. Icatu (Icatu) differentially expressed genes (DEGs) involved in the light reactions of photosynthesis, the Calvin cycle, and the photorespiration pathways. Homologs were identified through blastx and their involvement in the studied pathways was visualized through MapMan, according to the *A. thaliana* reference genome. Red represents up-regulated DEGs and blue represents down-regulated DEGs.

Table S9. Regulation patterns of *Coffea canephora* cv. CL153 (CL153) differentially expressed genes (DEGs) involved in the light reactions of photosynthesis, the Calvin cycle, and the photorespiration pathways. Homologs were identified through blastx and their involvement in the studied pathways was visualized through MapMan, according to the *A. thaliana* reference genome. Red represents up-regulated DEGs and blue represents down-regulated DEGs.

Table S10. Regulation patterns of drought-related differentially expressed genes (DEGs) *Coffea arabica* cv. Icatu (Icatu) and *Coffea canephora* cv. CL153 (CL153) plants grown under moderate water deficit (MWD) or severe water deficit (SWD) in comparison with well-watered (WW) plants, under ambient air 380 μL L^{-1} [CO_2] (aCO_2) or elevated 700 μL L^{-1} [CO_2] (eCO_2), at 25/20 °C (day/night). Red represents up-regulated DEGs and blue represents down-regulated DEGs.

Table S11. Significantly (g:SCS < 0.01) enriched gene ontology (GO) terms of the three main categories—Biological Process (GO:BP), Molecular Function (GO:MF), and Cellular Component (GO:CC)—among up- and down-regulated differentially expressed genes (DEGs) found in *Coffea arabica* cv. Icatu (Icatu) plants grown under moderate water deficit (MWD) or severe water deficit (SWD), in comparison with well-watered control plants, under ambient air 380 μL L^{-1} [CO_2] (aCO_2) or elevated 700 μL L^{-1} [CO_2] (eCO_2), at 25/20 °C (day/night). DEGs were ranked by increasing log2 fold-change (FC) and an over-representation analysis (ORA) was performed using gProfiler, against *C. canephora* functional annotation. Counts indicate the total number of DEGs annotated with each GO term.

Table S12. Significantly (g:SCS < 0.01) enriched gene ontology (GO) terms of the three main categories—Biological Process (GO:BP), Molecular Function (GO:MF), and Cellular Component (GO:CC)—among up- and down-regulated differentially expressed genes (DEGs) found in *Coffea canephora* cv. CL153 (CL153) plants grown under moderate water deficit (MWD) or severe water deficit (SWD), in comparison with well-watered control plants, under ambient air 380 μL L^{-1} [CO_2] (aCO_2) or elevated 700 μL L^{-1} [CO_2] (eCO_2), at 25/20 °C (day/night). DEGs were ranked by increasing log2 fold-change (FC) and an over-representation analysis (ORA) was performed using gProfiler, against *C. arabica* functional annotation. Counts indicate the total number of DEGs annotated with each GO term
