# Supplementary material for: Overexpression of Water-Responsive Genes Promoted by Elevated CO2 Reduces ROS and Enhances Drought Tolerance in Coffea Species
Source: Int J Mol Sci. 2023 Feb 6;24(4):3210. doi: 10.3390/ijms24043210 (PMC9966387; doi:10.3390/ijms24043210)
Supplement: Supplementary file 1 [file ijms-24-03210-s001.zip › Table_S1.pdf]

Table S1. Primers used in this study for qRT-PCR. ASPG1: Aspartic Protease in Guard Cell 1; GMPM1: 18 kDa seed maturation; PP2C51: protein phosphatase 2C 51-like; LEADC3: late embryogenesis abundant protein Dc3-like; DH1a: Dehydrin DH1a; ATHB22: homeobox leucine zipper; SUS2: sucrose synthase 2-like; PIP2: aquaporin PIP2-2-like; XTH6: xyloglucan endotransglucosylase/hydrolase protein 6; GOLS2: galactinol synthase 2-like; CuSOD1: Superoxide dismutase [Cu-Zn]; APXChl: Chloroplast ascorbate peroxidase.

**Primer Name**	**Sequence (5′–3′)**
ASPG1-F	TTTCCTGCAGGTTGTGGATGTTAG
ASPG1-R	TCCCCCGGGTATTTCCGGCGAAG
GMPM1-F	CTGGAACCGCCACGTATTCTCC
GMPM1-R	TTAAGTACCCCCAGTCCCATAC
PP2C51-F	CCGAGTACGGAAGCTGCAAGAAGT
PP2C51-R	GCAGCGATAGTGGCAGGGAAACAA
LEADC3_F	CTACGGCTACTGTCAACGATGTGAA
LEADC3_R	ATGGGTAAAGAATGGCTGTATCCCT
DH1a-F	CGGTCACAAGGAGGCTCAA
DH1a-R	GTATCCAGGAGCTGCAGTAGCA
ATHB22-F	AGTTGGAGCGAAGATAAGTTC
ATHB22-R	TCACCGGAAGGCATAAATCTAC
SUS2-F	ACTCTGCGGCAATG TAAACT
SUS2-R	AGACTGCCGCGGAGACCAGA
PIP2-F	ATGTCACATACGGCGGAG
PIP2-R	TTGGGATCAGTGGCAGAG
XTH6-F	CGGTCACCGCTTTCTTTTCAGTT
XTH6-R	CAAGAACTCAAAATCTAGCTCGT
GOLS2-F	ATGGGATCGATGGAAATGAACTT
GOL2-R	ACTTCCTGCCAAGAATGTCAC
CuSOD1-F	CCCTTGGAGACACAACGAAT
CuSOD1-R	GGCAGTACCATCTTGACCA
APX_{Chl}-F	CACCTGCTGCTCATTTACG
APX_{Chl}-R	GACCTTCCCAATGTGTGTG
