# Supplementary material for: Overexpression of Water-Responsive Genes Promoted by Elevated CO2 Reduces ROS and Enhances Drought Tolerance in Coffea Species
Source: Int J Mol Sci. 2023 Feb 6;24(4):3210. doi: 10.3390/ijms24043210 (PMC9966387; doi:10.3390/ijms24043210)
Supplement: Supplementary file 1 [file ijms-24-03210-s001.zip › Table_S11.pdf]

Table S11. Significantly (g:SCS < 0.01) enriched Gene Ontology (GO) terms of the three main categories – Biological Process (GO:BP), Molecular Function (GO:MF) and Cellular Component (GO:CC) – among up- and down-regulated Differentially Expressed Genes (DEGs) found in *Coffea arabica* cv. Icatu (Icatu) plants grown under moderate water deficit (MWD) or severe water deficit (SWD), in comparison with well-watered control plants, under ambient air 380 μL L^{-1} [CO_2] (aCO_2) or elevated 700 μL L^{-1} [CO_2] (eCO_2), at 25/20 °C (day/night). DEGs were ranked by increasing log2 fold-change (FC) and an Over-representation analysis (ORA) was performed using gProfiler, against *C. canephora* functional annotation. Counts indicate the total number of DEGs annotated with each GO term.

[CO_2]	Water	Category	Term ID	Description	DEGs	FDR
**aCO_2**	**MWD**			**Down**		
		**BP**	GO:0071555	cell wall organization	26	2.60E-17
			GO:0005975	carbohydrate metabolic process	23	2.43E-08
			GO:0045490	pectin catabolic process	6	1.28E-05
			GO:0042744	hydrogen peroxide catabolic process	8	3.54E-05
			GO:0016036	cellular response to phosphate starvation	4	1.63E-04
			GO:0009813	flavonoid biosynthetic process	3	1.73E-04
			GO:0006878	cellular copper ion homeostasis	4	1.94E-04
			GO:0006979	response to oxidative stress	8	1.77E-03
		**CC**	GO:0005618	cell wall	21	1.12E-21
			GO:0048046	apoplast	15	5.87E-14
			GO:0005576	extracellular region	25	5.96E-13
			GO:0031225	anchored component of membrane	8	1.84E-05
		**MF**	GO:0004553	hydrolase activity hydrolyzing O-glycosyl compounds	16	8.86E-13
			GO:0016762	xyloglucosyl transferase activity	7	2.87E-07

		GO:0030145	manganese ion binding	7	2.84E-06
		GO:0004190	aspartic-type endopeptidase activity	11	3.64E-05
		GO:0016210	naringenin-chalcone synthase activity	3	7.23E-05
		GO:0004601	peroxidase activity	8	1.54E-04
		GO:0005375	copper ion transmembrane transporter activity	4	1.94E-04
		GO:0004857	enzyme inhibitor activity	7	5.30E-04
		GO:0016788	hydrolase activity acting on ester bonds	6	6.71E-04
		GO:0030570	pectate lyase activity	3	1.14E-03
			**Up**		
	**BP**	GO:0009737	response to abscisic acid	2	1.05E-03
	**MF**	GO:0000981	DNA-binding transcription factor activity RNA polymerase II-specific	3	8.79E-04
		GO:0004869	cysteine-type endopeptidase inhibitor activity	3	4.87E-03
**SWD**			**Down**		
	**BP**	GO:0015979	photosynthesis	33	1.13E-23
		GO:0071555	cell wall organization	38	1.57E-16
		GO:0045490	pectin catabolic process	8	1.97E-08
		GO:0009765	photosynthesis light harvesting	11	2.22E-07
		GO:0005975	carbohydrate metabolic process	41	2.15E-06
		GO:0015995	chlorophyll biosynthetic process	9	2.92E-04
		GO:0018298	protein-chromophore linkage	10	1.54E-03
		GO:0006633	fatty acid biosynthetic process	14	9.58E-03
	**CC**	GO:0005618	cell wall	18	9.03E-16

	GO:0009523	photosystem II	17	3.89E-13
	GO:0009522	photosystem I	16	1.73E-12
	GO:0005576	extracellular region	30	1.22E-10
	GO:0048046	apoplast	11	5.32E-07
	GO:0016021	integral component of membrane	351	6.38E-07
	GO:0009507	chloroplast	20	9.65E-03
**MF**	GO:0004857	enzyme inhibitor activity	16	7.15E-11
	GO:0016760	cellulose synthase (UDP-forming) activity	13	7.82E-08
	GO:0016168	chlorophyll binding	11	2.22E-07
	GO:0004553	hydrolase activity hydrolyzing O-glycosyl compounds	29	3.27E-07
	GO:0008171	O-methyltransferase activity	10	1.98E-06
	GO:0030599	pectinesterase activity	6	4.24E-06
	GO:0045330	aspartyl esterase activity	6	4.24E-06
	GO:0015267	channel activity	11	3.65E-05
	GO:0030145	manganese ion binding	8	4.86E-05
	GO:0102337	3-oxo-cerotoyl-CoA synthase activity	9	1.54E-04
	GO:0102336	3-oxo-arachidoyl-CoA synthase activity	9	1.54E-04
	GO:0102756	very-long-chain 3-ketoacyl-CoA synthase activity	9	1.54E-04
	GO:0102338	3-oxo-lignoceronyl-CoA synthase activity	9	1.54E-04
	GO:0004190	aspartic-type endopeptidase activity	15	5.39E-04
	GO:0042132	fructose 16-bisphosphate 1-phosphatase activity	4	6.98E-03
		**Up**		

		BP	GO:0019953	sexual reproduction	3	8.77E-07
			GO:0009627	systemic acquired resistance	3	1.80E-03
		MF	GO:0045735	nutrient reservoir activity	4	4.94E-04
			GO:0005504	fatty acid binding	2	9.32E-03
**eCO_2**	**SWD**			**Down**		
		BP	GO:0015979	photosynthesis	28	1.57E-13
			GO:0005975	carbohydrate metabolic process	68	5.56E-11
			GO:0071555	cell wall organization	35	3.43E-10
			GO:0006817	phosphate ion transport	48	1.56E-07
			GO:0006633	fatty acid biosynthetic process	23	9.24E-07
			GO:0015995	chlorophyll biosynthetic process	12	1.60E-05
			GO:0009765	photosynthesis light harvesting	10	3.11E-05
			GO:2000123	positive regulation of stomatal complex development	2	1.42E-04
			GO:0045490	pectin catabolic process	9	5.29E-04
			GO:0042744	hydrogen peroxide catabolic process	7	1.72E-03
			GO:0018298	protein-chromophore linkage	12	1.27E-02
			GO:0006979	response to oxidative stress	7	2.87E-02
			GO:0000226	microtubule cytoskeleton organization	4	3.21E-02
			GO:0007017	microtubule-based process	9	4.07E-02
		CC	GO:0016021	integral component of membrane	606	1.51E-21
			GO:0005618	cell wall	18	1.07E-12

	GO:0005576	extracellular region	45	4.80E-12
	GO:0005886	plasma membrane	86	6.54E-11
	GO:0009522	photosystem I	15	3.70E-08
	GO:0009523	photosystem II	13	1.50E-05
	GO:0005874	microtubule	24	1.78E-04
	GO:0048046	apoplast	10	1.94E-04
	GO:0031225	anchored component of membrane	13	3.65E-04
	GO:0019898	extrinsic component of membrane	9	4.78E-04
**MF**	GO:0004553	hydrolase activity hydrolyzing O-glycosyl compounds	33	6.62E-12
	GO:0022857	transmembrane transporter activity	62	4.54E-10
	GO:0004672	protein kinase activity	88	9.42E-08
	GO:0016747	transferase activity transferring acyl groups other than amino-acyl groups	15	1.84E-06
	GO:0008171	O-methyltransferase activity	12	7.97E-06
	GO:0004857	enzyme inhibitor activity	14	3.04E-05
	GO:0016168	chlorophyll binding	10	3.11E-05
	GO:0005199	structural constituent of cell wall	5	1.24E-04
	GO:0016787	hydrolase activity	26	4.71E-04
	GO:0016762	xyloglucosyl transferase activity	8	8.49E-04
	GO:0004097	catechol oxidase activity	4	1.06E-03
	GO:0102345	3-hydroxy-lignoceroyl-CoA dehydratase activity	4	2.78E-03
	GO:0102344	3-hydroxy-behenoyl-CoA dehydratase activity	4	2.78E-03

	GO:0102343	3-hydroxy-arachidoyl-CoA dehydratase activity	4	2.78E-03
	GO:0102158	very-long-chain 3-hydroxyacyl-CoA dehydratase activity	4	2.78E-03
	GO:0030145	manganese ion binding	6	3.74E-03
	GO:0046983	protein dimerization activity	24	4.80E-03
	GO:0005200	structural constituent of cytoskeleton	8	4.84E-03
	GO:0004601	peroxidase activity	7	5.00E-03
	GO:0004190	aspartic-type endopeptidase activity	15	5.48E-03
	GO:0030570	pectate lyase activity	2	1.64E-02
	GO:0016630	protochlorophyllide reductase activity	3	1.87E-02
	GO:0016717	oxidoreductase activity acting on paired donors with oxidation of a pair of donors resulting in the reduction of molecular oxygen to two molecules of water	5	1.90E-02
	GO:0042132	fructose 16-bisphosphate 1-phosphatase activity	4	2.52E-02
	GO:0016788	hydrolase activity acting on ester bonds	4	3.92E-02
	GO:0030599	pectinesterase activity	6	4.98E-02
	GO:0045330	aspartyl esterase activity	6	4.98E-02
		**Up**		
**BP**	GO:0019953	sexual reproduction	3	3.05E-08
	GO:0009627	systemic acquired resistance	3	1.74E-05
	GO:0009415	response to water	3	7.78E-04
	GO:0000272	polysaccharide catabolic process	4	7.80E-03
	GO:0006562	proline catabolic process	2	3.23E-02

	GO:0005975	carbohydrate metabolic process	17	3.45E-02
**CC**	GO:0005576	extracellular region	3	2.92E-03
	GO:0016602	CCAAT-binding factor complex	3	4.24E-02
**MF**	GO:0004869	cysteine-type endopeptidase inhibitor activity	5	4.80E-06
	GO:0010427	abscisic acid binding	8	1.40E-05
	GO:0038023	signaling receptor activity	8	1.40E-05
	GO:0016620	oxidoreductase activity acting on the aldehyde or oxo group of donors NAD or NADP as acceptor	7	1.73E-03
	GO:0008194	UDP-glycosyltransferase activity	17	3.35E-03
	GO:0016161	beta-amylase activity	4	7.80E-03
	GO:0102229	amylopectin maltohydrolase activity	4	7.80E-03
	GO:0043565	sequence-specific DNA binding	16	8.41E-03
	GO:0004476	mannose-6-phosphate isomerase activity	2	2.19E-02
	GO:0020037	heme binding	21	2.64E-02
	GO:0009055	electron transfer activity	8	2.82E-02
	GO:0004657	proline dehydrogenase activity	2	3.23E-02
	GO:0003700	DNA-binding transcription factor activity	24	3.90E-02
