# Supplementary material for: Overexpression of Water-Responsive Genes Promoted by Elevated CO2 Reduces ROS and Enhances Drought Tolerance in Coffea Species
Source: Int J Mol Sci. 2023 Feb 6;24(4):3210. doi: 10.3390/ijms24043210 (PMC9966387; doi:10.3390/ijms24043210)
Supplement: Supplementary file 1 [file ijms-24-03210-s001.zip › Table_S12.pdf]

Table S12. Significantly (g:SCS < 0.01) enriched Gene Ontology (GO) terms of the three main categories – Biological Process (GO:BP), Molecular Function (GO:MF) and Cellular Component (GO:CC) – among up- and down-regulated Differentially Expressed Genes (DEGs) found in *Coffea canephora* cv. CL153 (CL153) plants grown under moderate water deficit (MWD) or severe water deficit (SWD), in comparison with well-watered control plants, under ambient air 380 μL L^{-1} [CO_2] (aCO_2) or elevated 700 μL L^{-1} [CO_2] (eCO_2), at 25/20 °C (day/night). DEGs were ranked by increasing log2 fold-change (FC) and an Over-representation analysis (ORA) was performed using gProfiler, against *C. arabica* functional annotation. Counts indicate the total number of DEGs annotated with each GO term.

[CO_2]	Water	Category	ID	Description	Counts	FDR
aCO_2	MWD			Down		
		BP	GO:0071555	cell wall organization	15	4.57E-06
		BP	GO:0045229	external encapsulating structure organization	15	7.54E-06
		BP	GO:0009664	plant-type cell wall organization	7	4.66E-05
		BP	GO:0071554	cell wall organization or biogenesis	15	5.51E-05
		BP	GO:0071669	plant-type cell wall organization or biogenesis	7	5.41E-04
		BP	GO:0009733	response to auxin	11	8.98E-04
		BP	GO:0006810	transport	46	5.04E-03
		BP	GO:0051234	establishment of localization	46	6.10E-03
		BP	GO:0030001	metal ion transport	10	7.31E-03
		BP	GO:0051179	localization	46	8.27E-03
		BP	GO:0055085	transmembrane transport	30	9.75E-03
		BP	GO:0055114	obsolete oxidation-reduction process	7	2.76E-03
		CC	GO:0005618	cell wall	13	3.00E-07
		CC	GO:0030312	external encapsulating structure	13	3.87E-07
		CC	GO:0005576	extracellular region	16	9.51E-05
		CC	GO:0071944	cell periphery	23	1.49E-03
		MF	GO:0022857	transmembrane transporter activity	31	4.10E-03
		MF	GO:0005215	transporter activity	31	6.46E-03
				Up		
		MF	GO:0003700	DNA-binding transcription factor activity	16	1.16E-03
		MF	GO:0016491	oxidoreductase activity	7	2.73E-03

	MF	GO:0050660	flavin adenine dinucleotide binding	3	3.90E-03
	MF	GO:0140110	transcription regulator activity	16	4.95E-03
	MF	GO:0004190	aspartic-type endopeptidase activity	7	8.00E-03
	MF	GO:0070001	aspartic-type peptidase activity	7	8.00E-03
SWD	Down				
	BP	GO:0015979	photosynthesis	44	2.49E-23
	BP	GO:0019684	photosynthesis light reaction	20	3.61E-10
	BP	GO:0005976	polysaccharide metabolic process	22	9.70E-10
	BP	GO:0009765	photosynthesis light harvesting	12	6.53E-09
	BP	GO:0071555	cell wall organization	21	6.96E-09
	BP	GO:0045229	external encapsulating structure organization	21	1.35E-08
	BP	GO:0071554	cell wall organization or biogenesis	24	1.67E-08
	BP	GO:0018298	protein-chromophore linkage	13	1.91E-07
	BP	GO:0009664	plant-type cell wall organization	10	3.19E-07
	BP	GO:0071669	plant-type cell wall organization or biogenesis	11	9.14E-07
	BP	GO:0006468	protein phosphorylation	130	3.04E-06
	BP	GO:0044264	cellular polysaccharide metabolic process	13	1.22E-05
	BP	GO:0044042	glucan metabolic process	12	1.80E-05
	BP	GO:0006073	cellular glucan metabolic process	12	1.80E-05
	BP	GO:0009733	response to auxin	22	2.81E-05
	BP	GO:0000272	polysaccharide catabolic process	11	4.98E-05
	BP	GO:0030243	cellulose metabolic process	8	1.46E-04
	BP	GO:0010393	galacturonan metabolic process	10	1.53E-04
	BP	GO:0045488	pectin metabolic process	10	1.53E-04
	BP	GO:0016310	phosphorylation	140	1.85E-04
	BP	GO:0044262	cellular carbohydrate metabolic process	14	3.10E-04
	BP	GO:0005975	carbohydrate metabolic process	83	3.56E-04
	BP	GO:0042546	cell wall biogenesis	9	3.63E-04
	BP	GO:0051273	beta-glucan metabolic process	8	5.91E-04
	BP	GO:0009734	auxin-activated signaling pathway	18	6.64E-04
	BP	GO:0071365	cellular response to auxin stimulus	18	8.80E-04

BP	GO:0009719	response to endogenous stimulus	25	1.17E-03
BP	GO:0009725	response to hormone	25	1.17E-03
BP	GO:0030244	cellulose biosynthetic process	13	1.78E-03
BP	GO:0045490	pectin catabolic process	8	1.85E-03
BP	GO:0034637	cellular carbohydrate biosynthetic process	25	3.49E-03
BP	GO:0008150	biological_process	879	4.60E-03
BP	GO:0010410	hemicellulose metabolic process	6	5.08E-03
BP	GO:0006091	generation of precursor metabolites and energy	38	5.33E-03
BP	GO:0010033	response to organic substance	26	9.61E-03
CC	GO:0009521	photosystem	32	1.72E-20
CC	GO:0009522	photosystem I	21	1.74E-16
CC	GO:0009579	thylakoid	51	4.94E-16
CC	GO:0034357	photosynthetic membrane	44	1.37E-15
CC	GO:0009523	photosystem II	23	3.89E-12
CC	GO:0042651	thylakoid membrane	37	4.62E-11
CC	GO:0071944	cell periphery	60	7.67E-09
CC	GO:0031224	intrinsic component of membrane	432	1.10E-08
CC	GO:0016020	membrane	487	1.20E-08
CC	GO:0016021	integral component of membrane	430	2.00E-08
CC	GO:0005618	cell wall	21	4.32E-08
CC	GO:0055035	plastid thylakoid membrane	30	4.37E-08
CC	GO:0009535	chloroplast thylakoid membrane	32	4.97E-08
CC	GO:0031976	plastid thylakoid	32	1.29E-07
CC	GO:0030312	external encapsulating structure	20	4.45E-07
CC	GO:0009534	chloroplast thylakoid	31	5.97E-07
CC	GO:0005576	extracellular region	44	1.42E-06
CC	GO:0005886	plasma membrane	75	7.16E-05
CC	GO:0009538	photosystem I reaction center	5	1.27E-03
CC	GO:0048046	apoplast	10	1.73E-03
MF	GO:0003824	catalytic activity	760	1.00E-12
MF	GO:0016168	chlorophyll binding	12	2.73E-09

		MF	GO:0016740	transferase activity	321	1.23E-05
		MF	GO:0004672	protein kinase activity	125	2.86E-05
		MF	GO:0008194	UDP-glycosyltransferase activity	57	3.56E-05
		MF	GO:0016798	hydrolase activity acting on glycosyl bonds	36	5.82E-05
		MF	GO:0004553	hydrolase activity hydrolyzing O-glycosyl compounds	34	9.25E-05
		MF	GO:0016758	transferase activity transferring hexosyl groups	67	1.00E-04
		MF	GO:0016682	oxidoreductase activity acting on diphenols and related substances as donors oxygen as acceptor	6	1.33E-04
		MF	GO:0016679	oxidoreductase activity acting on diphenols and related substances as donors	6	1.65E-04
		MF	GO:0016773	phosphotransferase activity alcohol group as acceptor	127	1.19E-03
		MF	GO:0008146	sulfotransferase activity	8	1.48E-03
		MF	GO:0016759	cellulose synthase activity	10	1.78E-03
		MF	GO:0016760	cellulose synthase (UDP-forming) activity	10	1.78E-03
		MF	GO:0016757	transferase activity transferring glycosyl groups	73	4.04E-03
		MF	GO:0016301	kinase activity	132	5.14E-03
		MF	GO:0004097	catechol oxidase activity	3	5.44E-03
		MF	GO:0046527	glucosyltransferase activity	17	6.07E-03
				Up		
		MF	GO:0016491	oxidoreductase activity	46	3.21E-03
		MF	GO:0050660	flavin adenine dinucleotide binding	3	9.00E-03
eCO_2	MWD			Down		
		BP	GO:0009664	plant-type cell wall organization	3	4.15E-03
	SWD			Down		
		BP	GO:0015979	photosynthesis	44	1.77E-27
		BP	GO:0006091	generation of precursor metabolites and energy	43	3.01E-10
		BP	GO:0019684	photosynthesis light reaction	18	3.15E-09
		BP	GO:0009765	photosynthesis light harvesting	9	6.39E-06
		BP	GO:0005975	carbohydrate metabolic process	77	9.53E-06
		BP	GO:0071555	cell wall organization	16	2.62E-05
		BP	GO:0045229	external encapsulating structure organization	16	4.04E-05
		BP	GO:0071554	cell wall organization or biogenesis	17	1.18E-04

BP	GO:0009664	plant-type cell wall organization	7	1.60E-04
BP	GO:0018298	protein-chromophore linkage	11	2.30E-04
BP	GO:0010258	NADH dehydrogenase complex (plastoquinone) assembly	4	1.06E-03
BP	GO:0072330	monocarboxylic acid biosynthetic process	17	1.79E-03
BP	GO:0071669	plant-type cell wall organization or biogenesis	7	2.05E-03
BP	GO:0008610	lipid biosynthetic process	31	2.22E-03
BP	GO:0010065	primary meristem tissue development	3	4.34E-03
BP	GO:0006633	fatty acid biosynthetic process	15	5.01E-03
BP	GO:0008150	biological_process	614	5.35E-03
BP	GO:0010207	photosystem II assembly	5	5.92E-03
CC	GO:0009579	thylakoid	61	6.33E-33
CC	GO:0034357	photosynthetic membrane	52	4.42E-29
CC	GO:0042651	thylakoid membrane	48	3.18E-26
CC	GO:0009521	photosystem	31	5.92E-23
CC	GO:0009535	chloroplast thylakoid membrane	42	8.20E-22
CC	GO:0031976	plastid thylakoid	41	4.26E-20
CC	GO:0009507	chloroplast	95	2.21E-19
CC	GO:0009534	chloroplast thylakoid	40	4.22E-19
CC	GO:0055035	plastid thylakoid membrane	38	4.34E-19
CC	GO:0009536	plastid	92	1.68E-17
CC	GO:0009523	photosystem II	24	3.42E-16
CC	GO:0009522	photosystem I	17	1.28E-14
CC	GO:0005576	extracellular region	31	1.48E-07
CC	GO:0005618	cell wall	16	2.13E-07
CC	GO:0009654	photosystem II oxygen evolving complex	11	3.77E-07
CC	GO:0098796	membrane protein complex	38	8.68E-07
CC	GO:0030312	external encapsulating structure	15	2.55E-06
CC	GO:0009941	chloroplast envelope	28	8.49E-06
CC	GO:1990204	oxidoreductase complex	14	2.62E-05
CC	GO:0009538	photosystem I reaction center	5	4.36E-05
CC	GO:0009526	plastid envelope	26	8.52E-05

CC	GO:0016020	membrane	363	5.65E-04
CC	GO:0031984	organelle subcompartment	53	9.88E-04
CC	GO:0071944	cell periphery	25	5.56E-03
MF	GO:0016168	chlorophyll binding	9	3.63E-06
MF	GO:0016798	hydrolase activity acting on glycosyl bonds	24	1.23E-05
MF	GO:0004553	hydrolase activity hydrolyzing O-glycosyl compounds	22	5.81E-05
MF	GO:0005200	structural constituent of cytoskeleton	6	5.46E-03
MF	GO:0003824	catalytic activity	477	6.85E-03
		Up		
BP	GO:2001141	regulation of RNA biosynthetic process	56	2.62E-04
BP	GO:1903506	regulation of nucleic acid-templated transcription	56	2.62E-04
BP	GO:0006355	regulation of transcription DNA-templated	56	2.62E-04
BP	GO:2000112	regulation of cellular macromolecule biosynthetic process	59	2.76E-04
BP	GO:0055114	obsolete oxidation-reduction process	29	2.91E-04
BP	GO:0010556	regulation of macromolecule biosynthetic process	59	3.01E-04
BP	GO:0051252	regulation of RNA metabolic process	56	4.40E-04
BP	GO:0031326	regulation of cellular biosynthetic process	59	4.56E-04
BP	GO:0009889	regulation of biosynthetic process	59	5.37E-04
BP	GO:0031323	regulation of cellular metabolic process	65	8.57E-04
BP	GO:0019219	regulation of nucleobase-containing compound metabolic process	56	8.58E-04
BP	GO:0080090	regulation of primary metabolic process	63	1.01E-03
BP	GO:0051171	regulation of nitrogen compound metabolic process	62	1.25E-03
BP	GO:0042742	defense response to bacterium	10	3.23E-03
BP	GO:0009617	response to bacterium	10	3.23E-03
BP	GO:0019222	regulation of metabolic process	68	4.01E-03
BP	GO:0010468	regulation of gene expression	60	4.55E-03
BP	GO:0006351	transcription DNA-templated	58	4.72E-03
BP	GO:0050832	defense response to fungus	9	5.74E-03
BP	GO:0050789	regulation of biological process	93	5.77E-03
BP	GO:0006952	defense response	32	6.02E-03
BP	GO:0097659	nucleic acid-templated transcription	58	6.04E-03

BP	GO:0032774	RNA biosynthetic process	58	6.48E-03
BP	GO:0060255	regulation of macromolecule metabolic process	65	7.02E-03
MF	GO:0140110	transcription regulator activity	36	2.48E-04
MF	GO:0016491	oxidoreductase activity	29	3.21E-04
MF	GO:0003700	DNA-binding transcription factor activity	31	2.80E-03
MF	GO:0003824	catalytic activity	211	4.71E-03
