# Supplementary material for: Overexpression of Water-Responsive Genes Promoted by Elevated CO2 Reduces ROS and Enhances Drought Tolerance in Coffea Species
Source: Int J Mol Sci. 2023 Feb 6;24(4):3210. doi: 10.3390/ijms24043210 (PMC9966387; doi:10.3390/ijms24043210)
Supplement: Supplementary file 1 [file ijms-24-03210-s001.zip › Table_S2.pdf]

Table S2. Summary of sequencing and mapping of reads from *Coffea arabica* cv. Icatu (Icatu) and *C. canephora* cv. CL153 (CL153) grown under moderate water deficit (MWD) or severe water deficit (SWD), and control well-watered plants, either under ambient 380 μL L^{-1} [CO_2] (aCO_2) or elevated 700 μL L^{-1} [CO_2] (eCO_2) at 25/20 °C (day/night). Raw reads: number of reads obtained after sequencing. Uniquely mapped reads: number of reads aligned to a unique position. % Unique: proportion of reads aligned to a unique position compared to the number of raw reads. Multiple mapped reads: number of reads aligned to exons of several overlapping genes. % Multiple: proportion of reads aligned to exons of several overlapping genes compared to the number of raw reads. Unmapped reads: number of non-aligning reads. % Unmapped: proportion of non-aligning reads compared to the number of raw reads.

Genotype	[CO_2]	Water	Replicates	Raw reads	Uniquely mapped reads	% Unique	Multiple mapped reads	% Multiple	Unmapped reads	% Unmapped
CL153	aCO_2	WW	1A	21337820	17963999	84,19	1068388	5,01	2305433	10,8
			1B	19886216	17435088	87,67	594232	2,99	1856896	9,34
			1C	23700779	19903897	83,98	954339	4,03	2842543	11,99
		MWD	2A	19019627	16370474	86,07	648828	3,41	2000325	10,51
			2B	29050476	23285935	80,16	1276101	4,4	4488440	15,45
			2C	43271586	34861296	80,56	1870546	4,32	6539744	15,11
		SWD	3A	21944385	20038816	91,32	712260	3,25	1193309	5,44
			3B	25523224	22250344	87,18	1051098	4,12	2221782	8,71
			3C	21988087	17169084	78,08	859075	3,91	3959928	18,01
	eCO_2	WW	4A	22261883	16699464	75,01	1257695	5,65	4304724	19,34
			4B	20883146	16062375	76,92	944211	4,52	3876560	18,56
			4C	22273972	18149138	81,48	1071574	4,81	3053260	13,70
		MWD	5A	20298860	16791383	82,72	689625	3,4	2817852	13,88
			5B	27587772	22173217	80,37	1034732	3,75	4379823	15,88
			5D	27609922	22106658	80,07	908956	3,29	4594308	16,64
		SWD	6A	19825056	16053978	80,98	722511	3,64	3048567	15,37

			6B	16377814	12186531	74,41	630264	3,85	3561019	21,74
			6C	21411839	16674575	77,88	772276	3,61	3964988	18,52
Average				23569581	19232014	82	948151	4	3389417	14
Icatu	aCO_2	WW	7A	26528307	19715321	74,32	1834267	6,91	4978719	1,47
			7B	22009479	17253113	78,39	746742	3,4	4007205	18,2
			7C	18666431	15290046	81,91	1003935	5,38	2372450	12,7
		MWD	8A	20273543	16298660	80,39	1111168	5,48	2863715	14,13
			20B	19550984	15205721	77,77	928936	4,75	3416327	17,47
			20C	24311127	18052291	74,26	1097624	4,52	5161212	21,23
		SWD	9B	19857280	15543358	78,28	810497	4,08	3503425	17,64
			9C	21199998	15184071	71,62	1173187	5,54	4842740	22,84
			9E	34865625	26076327	74,79	2187875	6,28	6601423	18,93
	eCO_2	WW	10A	23205533	18854251	81,25	1449795	6,26	2898972	12,5
			10B	21050167	17086784	81,17	822286	3,91	3141097	14,92
			10C	20541822	16146299	78,6	1170402	5,69	3225121	15,7
		MWD	11B	22590411	16234957	71,87	2353705	10,42	4001749	17,71
			11C	22711749	16458232	72,47	1894644	8,34	4358873	19,19
			11D	22099401	16303417	73,77	1730672	7,83	4065312	18,4
		SWD	12B	19703228	15925812	80,83	1187218	6,02	2590198	13,14
			12C	17710079	13447037	75,93	1075593	6,07	3187449	17,99
			12D	17091236	14668143	85,82	664943	3,89	1758150	10,28
Average				21887022	16874658	77	1291305	6	3720785	16
