# Supplementary material for: Overexpression of Water-Responsive Genes Promoted by Elevated CO2 Reduces ROS and Enhances Drought Tolerance in Coffea Species
Source: Int J Mol Sci. 2023 Feb 6;24(4):3210. doi: 10.3390/ijms24043210 (PMC9966387; doi:10.3390/ijms24043210)
Supplement: Supplementary file 1 [file ijms-24-03210-s001.zip › Table_S3.pdf]

Table S3. Number of total expressed genes in *Coffea arabica* cv. Icatu (Icatu) and *C. canephora* cv. CL153 (CL153), number of differentially expressed genes (DEGs) detected by DESeq2 and edgeR, with log2 fold-change (FC) ≠ 0 and false discovery rate (FDR) < 0.01, and number of overlapping DEGs from both analyses (% DEGs relative to the average of genes expressed by treated and control plants). Plants were grown in three different water conditions, well-watered (control), moderate water deficit (MWD), and severe water deficit (SWD), under either ambient air 380 μL L^{-1} [CO_2] (aCO_2) or elevated 700 μL L^{-1} [CO_2] (eCO_2), at 25/20 °C (day/night). DEGs represent the number of significant genes found to be differently expressed between each stress water treatment and the control (respectively, MWD vs. WW and SWD vs. WW) [overlap % is the proportion of DEGs detected by both edgeR and DESeq2 related to the average of genes expressed by control and treated plants].

Genotype	Water	[CO2]	Expressed genes		DEGs					
					DESeq2	edgeR	Overlapped			
			Control	Test			Total	%	Up	Down
Icatu	MWD	aCO_2	29519	29960	714	1026	548	1.84	66	482
		eCO_2	31041	29223	47	227	35	0.12	27	8
	SWD	aCO_2	29519	29199	2259	2436	1673	5.70	313	1360
		eCO_2	31041	29222	3670	3810	2940	9.76	859	2081
CL153	MWD	aCO_2	20463	20001	953	1165	704	3.48	295	409
		eCO_2	19818	19912	71	284	47	0.24	17	30
	SWD	aCO_2	20463	20348	3251	3150	2576	12.62	725	1851
		eCO_2	19818	19558	2793	2691	2040	10.36	768	1272
