# Supplementary material for: Overexpression of Water-Responsive Genes Promoted by Elevated CO2 Reduces ROS and Enhances Drought Tolerance in Coffea Species
Source: Int J Mol Sci. 2023 Feb 6;24(4):3210. doi: 10.3390/ijms24043210 (PMC9966387; doi:10.3390/ijms24043210)
Supplement: Supplementary file 1 [file ijms-24-03210-s001.zip › Table_S8.pdf]

Table S8. Regulation pattern among *Arabidopsis thaliana* homologs of the differentially expressed genes (DEGs) of *Coffea arabica* cv. Icatu (Icatu), related to the light reactions of photosynthesis, the Calvin cycle, and the photorespiration pathways. Homologs were identified through blastx and their involvement in the studied pathways was visualized through MapMan, according to the *A. thaliana* reference genome.

**Gene ID**	**TAIR**	**Protein Name**	**Log2FC**		
			**MWDa vs WWa**	**SWDa vs WWa**	**SWDe vs WWe**
LOC113709680	at1g11860	aminomethyltransferase, mitochondrial			-1.63
LOC113709724	at4g04640	ATP synthase gamma chain, chloroplastic-like		-2.58	-1.75
LOC113688109	at5g54270	chlorophyll a-b binding protein 13, chloroplastic		-3.62	-2.82
LOC113743471	at2g05100	chlorophyll a-b binding protein 36, chloroplastic		-2.43	-2.10
LOC113742137	at3g47470	chlorophyll a-b binding protein 4, chloroplastic-like isoform X2		-2.90	-3.50
LOC113731692	at1g45474	chlorophyll a-b binding protein CP24 10A, chloroplastic-like		-2.79	-2.16
LOC113712883	at4g10340	chlorophyll a-b binding protein CP26, chloroplastic		-3.29	-3.26
LOC113710161	at1g29930	chlorophyll a-b binding protein of LHCII type 1-like		-4.11	-3.18
LOC113716585	at3g61470	chlorophyll a-b binding protein, chloroplastic-like		-1.91	-1.53
LOC113693755	at3g54050	fructose-1,6-bisphosphatase, chloroplastic-like		-3.16	-1.58
LOC113731210	at4g38970	fructose-bisphosphate aldolase 1, chloroplastic-like		-1.89	
LOC113743786	at4g26530	fructose-bisphosphate aldolase 5, cytosolic isoform X2		-2.72	
LOC113719543	at1g12900	glyceraldehyde-3-phosphate dehydrogenase A, chloroplastic-like		-2.43	-1.38
LOC113712439	at1g42970	glyceraldehyde-3-phosphate dehydrogenase B, chloroplastic		-2.19	

LOC113703085	at1g32470	glycine cleavage system H protein, mitochondrial-like			-1.69
LOC113723858	at2g45290	Low Quality protein: transketolase, chloroplastic-like	6.19	7.84	7.19
LOC113702187	at3g50820	oxygen-evolving enhancer protein 1, chloroplastic		-1.92	-1.41
LOC113689091	at1g06680	oxygen-evolving enhancer protein 2, chloroplastic-like		-2.18	-1.66
LOC113730850	at4g05180	oxygen-evolving enhancer protein 3-2, chloroplastic-like		-4.52	-3.80
LOC113690443	at1g56190	phosphoglycerate kinase, chloroplastic-like		-2.89	-1.80
LOC113716676	at1g14150	photosynthetic NDH subunit of lumenal location 2, chloroplastic		-2.35	-1.49
LOC113709767	at1g31330	photosystem I reaction center subunit III, chloroplastic-like		-2.47	-2.15
LOC113710419	at1g30380	photosystem I reaction center subunit psaK, chloroplastic		-2.47	
LOC113741817	at3g16140	photosystem I reaction center subunit VI, chloroplastic-like		-2.51	-1.94
LOC113732043	at4g12800	photosystem I reaction center subunit XI, chloroplastic-like		-2.12	
LOC113705554	at1g08380	photosystem I subunit O-like		-2.72	-2.82
LOC113743532	at1g79040	photosystem II 10 kDa polypeptide, chloroplastic-like		-1.93	-1.52
LOC113697312	at3g21055	photosystem II 5 kDa protein, chloroplastic-like		-2.74	
LOC113697312	at3g21055	photosystem II 5 kDa protein, chloroplastic-like			-2.15
LOC113716969	at1g67740	photosystem II core complex proteins psbY, chloroplastic-like		-1.89	
LOC113715630	at2g30570	photosystem II reaction center W protein, chloroplastic		-2.18	-1.69
LOC113689677	at1g03600	photosystem II repair protein PSB27-H1, chloroplastic-like			-1.51
LOC113692840	at1g20340	plastocyanin-like		-2.09	
LOC113698884	at3g04790	probable ribose-5-phosphate isomerase 3, chloroplastic		-1.88	
LOC113707851	at5g44520	probable ribose-5-phosphate isomerase 4, chloroplastic isoform X1		-2.59	
LOC113728229	at2g46820	protein Curvature Thylakoid 1B, chloroplastic		-3.49	-2.28

LOC113732998	at4g15510	psbP domain-containing protein 1, chloroplastic-like		-1.97	-1.55
LOC113712265	at1g76450	psbP domain-containing protein 3, chloroplastic isoform X2			-1.45
LOC113703724	at5g11450	psbP domain-containing protein 5, chloroplastic isoform X2			-1.84
LOC113739930	at3g55330	psbP-like protein 1, chloroplastic isoform X2		-2.33	
LOC113698165	at1g67090	ribulose bisphosphate carboxylase small chain SSU8, chloroplastic-like		-2.29	
LOC113732386	at2g39730	ribulose bisphosphate carboxylase/oxygenase activase, chloroplastic-like	3.80	3.94	3.77
LOC113742776	at5g61410	ribulose-phosphate 3-epimerase, chloroplastic			-1.38
LOC113695105	at1g60950	uncharacterized protein LOC113695105		-3.96	-5.91
LOC113698106	at5g36120	uncharacterized protein LOC113698106			-1.61
