# Supplementary material for: Overexpression of Water-Responsive Genes Promoted by Elevated CO2 Reduces ROS and Enhances Drought Tolerance in Coffea Species
Source: Int J Mol Sci. 2023 Feb 6;24(4):3210. doi: 10.3390/ijms24043210 (PMC9966387; doi:10.3390/ijms24043210)
Supplement: Supplementary file 1 [file ijms-24-03210-s001.zip › Table_S9.pdf]

Table S9. Regulation patterns of *Coffea canephora* cv. CL153 (CL153) Differentially Expressed Genes (DEGs) involved in the light reactions of photosynthesis, the Calvin cycle, and the photorespiration pathways. Homologs were identified through blastx and their involvement in the studied pathways was visualized through MapMan, according to the *A. thaliana* reference genome. Red represents up-regulated DEGs and blue represents down-regulated DEGs.

Gene ID	TAIR	Protein Name	Log_2FC		
			MWDa vs WWa	SWDa vs WWa	SWDe vs WWe
Cc09_g01490	at1g11860	Aminomethyltransferase, mitochondrial	-2.79	-3.36	-3.26
Cc10_g06080	at4g09650	ATP synthase delta chain, chloroplastic		-1.97	-2.26
Cc09_g01650	at4g04640	ATP synthase gamma chain, chloroplastic		-2.08	-2.16
Cc05_g09650	at1g29930	Chlorophyll a-b binding protein 1, chloroplastic		-4.50	-5.42
Cc05_g12720	at5g54270	Chlorophyll a-b binding protein 13, chloroplastic		-5.29	-3.98
Cc09_g09500	at2g05100	Chlorophyll a-b binding protein 36, chloroplastic		-3.32	-3.29
Cc09_g02010	at3g54890	Chlorophyll a-b binding protein 6A, chloroplastic		-4.01	
Cc05_g09930	at1g61520	Chlorophyll a-b binding protein 8, chloroplastic		-2.90	-2.30
Cc10_g16210	at4g10340	Chlorophyll a-b binding protein CP26, chloroplastic		-4.16	
Cc06_g01460	at3g08940	Chlorophyll a-b binding protein CP29.2, chloroplastic		-3.07	-2.83
Cc10_g04190	at1g45474	Chlorophyll a-b binding protein P4, chloroplastic	-3.98	-3.60	-3.39
Cc11_g16910	at3g61470	Chlorophyll a-b binding protein, chloroplastic		-3.01	-2.59
Cc06_g12480	at1g19150	Chlorophyll a-b binding protein, chloroplastic			-1.55
Cc04_g03070	at1g79870	D-isomer specific 2-hydroxyacid dehydrogenase family protein		-2.79	-3.62
Cc07_g10820	at1g20020	Ferredoxin--NADP reductase, leaf-type isozyme, chloroplastic		-2.13	-2.23
Cc02_g22810	at1g10960	Ferredoxin, chloroplastic			-1.86
Cc06_g01860	at3g54050	Fructose-1,6-bisphosphatase, chloroplastic		-2.37	-2.28
Cc05_g15430	at4g26530	Fructose-bisphosphate aldolase, cytoplasmic isozyme 1			-1.93
Cc11_g00610	at1g12900	Glyceraldehyde-3-phosphate dehydrogenase A, chloroplastic		-2.50	-2.17
Cc10_g00410	at1g42970	Glyceraldehyde-3-phosphate dehydrogenase B, chloroplastic		-2.80	-2.32

Cc09_g10170	at1g68010	Glycerate dehydrogenase			-1.64
Cc08_g02390	at1g32470	Glycine cleavage system H protein, mitochondrial			-2.75
Cc05_g15690	at1g55670	Hypothetical protein		-3.85	
Cc04_g02830	at3g16250	NDH-dependent cyclic electron flow 1		-1.90	-1.80
Cc07_g05350	at3g50820	Oxygen-evolving enhancer protein 1, chloroplastic		-2.08	-2.03
Cc05_g00840	at1g06680	Oxygen-evolving enhancer protein 2, chloroplastic		-2.47	-2.22
Cc02_g11770	at4g05180	Oxygen-evolving enhancer protein 3-2, chloroplastic		-4.20	-3.19
Cc05_g10170	at3g14420	Peroxisomal (S)-2-hydroxy-acid oxidase		-1.83	-1.84
Cc05_g16370	at1g56190	Phosphoglycerate kinase, cytosolic	-2.28	-2.40	-2.90
Cc08_g03960	at1g32060	Phosphoribulokinase, chloroplastic		-2.16	-1.85
Cc03_g03590	at1g03130	Photosystem I reaction center subunit II, chloroplastic		-2.71	-2.37
Cc09_g06610	at1g31330	Photosystem I reaction center subunit III, chloroplastic		-3.50	-3.05
Cc01_g17500	at2g20260	Photosystem I reaction center subunit IV A, chloroplastic		-2.11	-2.27
Cc09_g08490	at1g30380	Photosystem I reaction center subunit psaK, chloroplastic		-5.09	-3.83
Cc04_g03050	at3g16140	Photosystem I reaction center subunit VI, chloroplastic		-2.36	-2.60
Cc01_g15890	at4g12800	Photosystem I reaction center subunit XI, chloroplastic		-2.70	-2.55
Cc01_g08780	at1g08380	photosystem I subunit O		-4.74	
Cc05_g15930	at1g79040	Photosystem II 10 kDa polypeptide, chloroplastic		-2.57	-1.91
Cc06_g23280	at3g21055	Photosystem II 5 kDa protein, chloroplastic		-4.00	-3.25
Cc11_g16230	at1g67740	Photosystem II core complex proteins psbY, chloroplastic		-2.85	-2.64
Cc01_g18800	at2g30570	Photosystem II reaction center W protein, chloroplastic		-2.98	-2.89
Cc10_g01080	at1g20340	Plastocyanin, chloroplastic		-2.63	-2.70
Cc07_g19080	at5g36120	Predicted protein			-1.83
Cc02_g33910	at2g28605	Predicted protein (Fragment)			-2.67
Cc07_g10450	at4g38970	Probable fructose-bisphosphate aldolase 2, chloroplastic		-1.76	-2.23
Cc01_g03210	at3g04790	Probable ribose-5-phosphate isomerase			-1.52
Cc02_g35130	at4g15510	PsbP domain-containing protein 1, chloroplastic		-1.83	-1.55
Cc10_g00260	at1g76450	PsbP domain-containing protein 3, chloroplastic			-1.61

Cc08_g08290	at5g11450	PsbP domain-containing protein 5, chloroplastic			-2.60
Cc11_g12760	at1g14150	PsbQ-like 2			-1.72
Cc08_g01190	at1g12550	Putative Glyoxylate reductase		-3.76	-3.56
Cc09_g07140	at5g44520	Putative Probable ribose-5-phosphate isomerase			-1.89
Cc06_g02340	at5g02120	Putative uncharacterized protein			-2.07
Cc00_g15710	at1g67090	Ribulose bisphosphate carboxylase small chain SSU11A, chloroplastic	-2.23	-2.77	-2.95
Cc02_g07500	at2g39730	Ribulose bisphosphate carboxylase/oxygenase activase 1, chloroplastic		2.70	-2.10
Cc02_g06960	at3g55800	Sedoheptulose-1,7-bisphosphatase, chloroplastic		-2.08	-2.34
Cc05_g06750	at1g03600	Thylakoid lumenal protein At1g03610, chloroplastic		-2.20	-2.62
Cc02_g39980	at2g46820	Thylakoid membrane phosphoprotein 14 kDa, chloroplastic		-2.93	-2.78
Cc02_g11220	at3g60750	Transketolase, chloroplastic	4.89	5.28	
Cc02_g16530	at2g21170	Triosephosphate isomerase, chloroplastic		-1.57	-2.50
